# Subversion of Niche-Signalling Pathways in Colorectal Cancer: What Makes and Breaks the Intestinal Stem Cell

**DOI:** 10.3390/cancers13051000

**Published:** 2021-02-27

**Authors:** Nathalie Sphyris, Michael C. Hodder, Owen J. Sansom

**Affiliations:** 1 Cancer Research UK Beatson Institute, Garscube Estate, Switchback Road, Glasgow G61 1BD, UK; n.sphyris@beatson.gla.ac.uk (N.S.); mhodder@crai.com (M.C.H.); 2 Institute of Cancer Sciences, University of Glasgow, Garscube Estate, Switchback Road, Glasgow G61 1QH, UK

**Keywords:** intestinal stem cells (ISCs), intestinal stem cell (ISC) niche, colorectal cancer (CRC), cancer stem cells (CSCs), consensus molecular subtypes (CMS), Wnt, Notch, BMP, YAP, regeneration

## Abstract

**Simple Summary:**

The intestinal epithelium—a single-cell layer lining the luminal surface of the small and large intestine—comprises an array of highly specialized cell types that perform diverse digestive functions while also forming a protective barrier against potentially toxic gut contents. As such, the intestinal epithelium is barraged by multiple extraneous stresses and undergoes constant renewal to replenish lost or damaged cells. This perpetual renewal is orchestrated by LGR5^+^ stem cells in response to multiple convergent instructive signals, emanating from cells in the immediate vicinity, collectively termed the intestinal stem cell niche. In addition, reserve stem-like cells and/or more mature cell types can assume the stem cell mantle and replenish the injured epithelium, if LGR5^+^ stem cell function is compromised. Here, we discuss the niche signals that govern the stem cell state, and how these go awry in the development of colorectal cancer.

**Abstract:**

The intestinal epithelium fulfils pleiotropic functions in nutrient uptake, waste elimination, and immune surveillance while also forming a barrier against luminal toxins and gut-resident microbiota. Incessantly barraged by extraneous stresses, the intestine must continuously replenish its epithelial lining and regenerate the full gamut of specialized cell types that underpin its functions. Homeostatic remodelling is orchestrated by the intestinal stem cell (ISC) niche: a convergence of epithelial- and stromal-derived cues, which maintains ISCs in a multipotent state. Following demise of homeostatic ISCs post injury, plasticity is pervasive among multiple populations of reserve stem-like cells, lineage-committed progenitors, and/or fully differentiated cell types, all of which can contribute to regeneration and repair. Failure to restore the epithelial barrier risks seepage of toxic luminal contents, resulting in inflammation and likely predisposing to tumour formation. Here, we explore how homeostatic niche-signalling pathways are subverted in tumorigenesis, enabling ISCs to gain autonomy from niche restraints (“ISC emancipation”) and transform into cancer stem cells capable of driving tumour initiation, progression, and therapy resistance. We further consider the implications of the pervasive plasticity of the intestinal epithelium for the trajectory of colorectal cancer, the emergence of distinct molecular subtypes, the propensity to metastasize, and the development of effective therapeutic strategies.

## 1. Introduction

**Figure 1:**
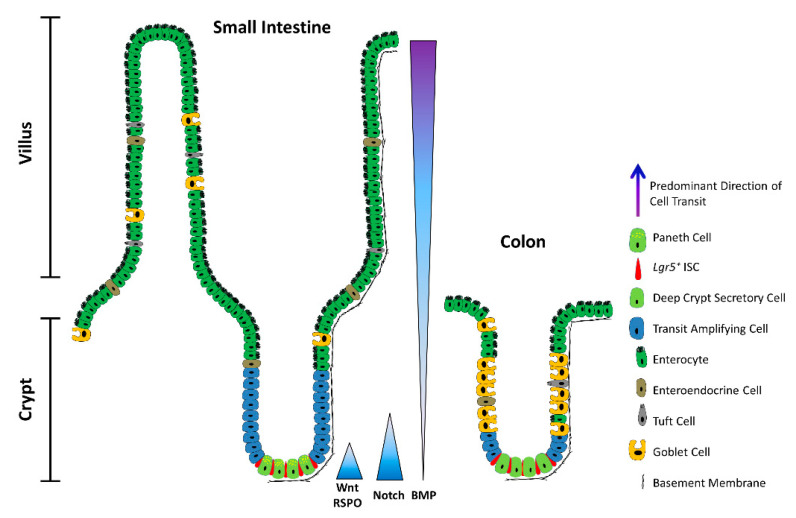
The architecture of the small intestine and the colon. Schematic depicting a longitudinal section of the intestinal mucosa. The mucosa of the small intestine extends finger-like projections (villi) into the gut lumen, which provide an increased surface area for optimal nutrient absorption. The villi are populated by mature, differentiated absorptive and secretory cell types, including absorptive enterocytes, hormone- and neurotransmitter-secreting enteroendocrine cells, mucus-secreting goblet cells, tuft cells, and microfold (M) cells (not shown). The mucosa surrounding the villi forms tubular invaginations into the lamina propria, called crypts, which serve as a protected reservoir of stem and progenitor cell populations. Notably, the epithelium of the colon is devoid of villi, with the crypts opening onto a flat mucosal surface, reflecting its role in waste compaction. To support homeostatic turnover, ISCs self-renew and give rise to short-lived transit-amplifying (TA) cells, which in turn beget lineage-restricted progenitors that differentiate into the mature cell types lining the villi. During their limited lifespan, intestinal epithelial cells migrate from the base of the crypt to the tip of the villus or the colonic surface, from where they are shed into the gut lumen and replaced by neighbouring cells. In contrast, Paneth cells are relatively long-lived, and migrate to the base of the crypt, where they secrete antimicrobial peptides and form a vital component of the ISC niche. Paneth cells are absent from the colon, but deep crypt secretory (DCS) cells may fulfil an equivalent role. Opposing gradients of morphogens specify intestinal-cell fate and differentiation along the vertical crypt axis: Wnt and Notch signalling prevail at the crypt base, whereas BMP transduction is highest near the lumen.

The single-layer epithelium lining the intestinal tract is integral to its functions in water and nutrient absorption, waste elimination, and immune surveillance while also forming a barrier against luminal toxins and gut-resident microbiota. To weather the barrage of chemical, pathogenic, and mechanical stresses posed by the digestive process, and counterbalance cell attrition, the intestine must continuously replenish its epithelial lining and regenerate the full gamut of specialized cell types that underpin its diverse functions. The homeostatic renewal of this epithelium is critically dependent on the sustained activity of multipotent intestinal stem cells (ISCs), residing within submucosal invaginations, called crypts. ISCs can self-renew while also giving rise to short-lived transit-amplifying (TA) cells, which in turn undergo successive rounds of cell division to generate multiple mature intestinal cell types (Figure 1). Broadly, differentiated intestinal cell lineages are specialized to perform either absorptive or secretory functions [1]. Absorptive enterocytes retrieve nutrients and water from luminal contents, whereas rare microfold (M) cells function in immune surveillance, delivering luminal antigens to underlying lymphoid structures (Peyer’s patches). Secretory lineages include multiple hormone- and neurotransmitter-secreting enteroendocrine cell types that regulate physiological responses to food intake and interface with the enteric nervous system, mucus-secreting goblet cells that fortify the host epithelium against mechanical stresses and luminal microorganisms, and chemosensory tuft cells that orchestrate type-2 immunity responses to helminth parasites and allergens [1].

Since aberrant ISC proliferation or, conversely, the failure to mobilize ISCs in response to injury is invariably detrimental, ISC activity is kept in check by the local milieu: the ISC niche. Set within the confines of the crypt base, the ISC niche is comprised of either Paneth cells in the small intestine or deep crypt secretory (DCS) cells in the colon, in addition to pericryptal fibroblasts, immune cells, endothelial cells, enteric neurocytes, extracellular matrix (ECM) components, and soluble cytokines and growth factors. Multiple converging niche-signalling pathways—primarily Wnt, Notch, and BMP—maintain ISCs in a multipotent state and fine-tune the balance between self-renewal and differentiation.

Due to the intricate three-dimensional structure of the intestinal crypt (Figure 1), resolving the nuances of the ISC niche using traditional two-dimensional cell cultures is impracticable. However, recent advances in transgenic animal modelling—particularly cell fate-mapping and lineage tracing—and the advent of organoid technologies and single-cell transcriptomics have illuminated the molecular mechanisms governing ISC behaviour. Here, we discuss the signalling pathways that constitute the ISC niche, and how their subversion may lead to what we term “ISC emancipation”, whereby ISCs gain autonomy from the niche. ISC emancipation arises when a mutation either negates ISC dependence on pro-proliferative and pro-survival niche signals, or enables ISCs to evade growth-inhibitory stimuli, permitting unbridled expansion of stem-like populations that spur tumour growth. Furthermore, we discuss emerging therapeutic approaches to curtail ISC emancipation and consider the implications of the pervasive plasticity of the intestinal epithelium for tumour initiation, progression, and treatment.

## 2. ISCs in a Nutshell

**Figure 2:**
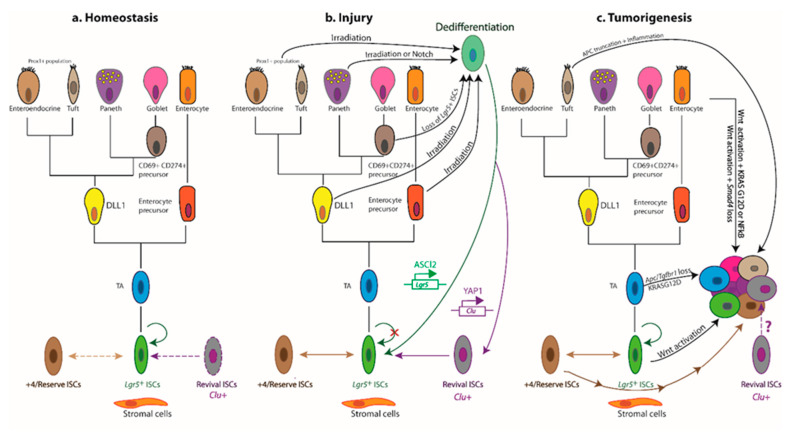
Cellular hierarchies and phenotypic plasticity in the intestinal epithelium during homeostasis, post-injury regeneration, and tumorigenesis. The dendrograms summarize key lineage relationships between *Lgr5*^+^ ISCs, transit-amplifying (TA) cells, lineage-committed precursors, terminally differentiated intestinal cell types, +4/reserve ISCs, and revival stem cells in different settings. (**a**) The homeostatic remodelling of the intestinal epithelium is orchestrated by niche signals that fine-tune the balance between *Lgr5*^+^ ISC self-renewal and differentiation. Revival stem cells are rare under homeostatic conditions (dashed boundary), and +4/reserve ISCs contribute only weakly to daily turnover during homeostasis (double-headed dashed arrow). (**b**) Following ablation of *Lgr5*^+^ ISCs post injury, +4/reserve ISCs can mobilize to replenish lost *Lgr5*^+^ ISCs and repopulate damaged crypts. Differentiated *Lgr5*^+^ progeny upregulate *Clu* in a YAP1-dependent manner, transiently serving as revival stem cells that can generate *Lgr5*^+^ ISCs de novo. Lineage-committed precursors and/or fully differentiated cells can dedifferentiate, revert to an *Lgr5*^+^ state, and regain stem-like traits. The transcription factor ASCL2 is critical for the ability of recent *Lgr5*^+^ ISC progeny to undergo dedifferentiation to an *Lgr5*^+^ state. Of note, *Clu*^+^ cells are distinct from the *Lgr5*^+^ and *Ascl2*^+^ populations. (**c**) Aberrant activation of Wnt signalling drives unbridled proliferation of *Lgr5*^+^ ISCs leading to intestinal hyperplasia. TA and differentiated cells, with hyperactive Wnt signalling and mutant KRAS^G12D^, may progress to malignancy in the context of TGFβ-receptor loss or inflammation (i.e., NFκB activation). Multiple +4/reserve ISCs can also initiate tumorigenesis, and tuft cells are readily transformed by inflammation following *Apc* loss. Whether revival stem cells can serve as tumour-initiating cells remains unclear. Solid arrows indicate the ability to dedifferentiate and revert to a stem-like state, or the susceptibility to transformation and hyperplastic progression. Reflexive arrows indicate the ability to self-renew. Double-headed solid arrows denote dynamic interconversion between indicated cell types. Note that, to date, goblet cell progenitors have not been lineage-traced.

Daily homeostatic turnover of the intestinal epithelium is orchestrated by crypt-base columnar cells, nestled between either Paneth or DCS cells at the crypt base in the small intestine and colon, respectively [2,3]. Decorated with the RSPO-receptor LGR5, which potentiates canonical Wnt/β-catenin signalling [4,5,6], these highly proliferative cells (hereafter *Lgr5*^+^ ISCs) exhibit the ability to self-renew and differentiate into all intestinal lineages in vitro and in vivo [7,8] and are tasked with the homeostatic renewal of the epithelium in both the small intestine and the colon [7] (Figure 2a). Yet, remarkably, the adult intestinal epithelium can fully recover following acute ablation of *Lgr5*-expressing cells [9], and conditional deletion of the *Lgr5* gene does not visibly perturb crypt architecture [4]. Together, these findings bring forth the redundancy of *Lgr5*^+^ ISCs for homeostasis and suggest that other cell types can compensate for their deficiency in this setting. Nevertheless, sustained depletion of *Lgr5*^+^ ISCs severely compromises the regenerative response to radiation-induced damage [10], suggesting that any compensatory cell types, deployed post injury, must first repopulate the *Lgr5*^+^ compartment prior to reconstituting the lost epithelium. Indeed, multiple putative reserve ISC pools have been proposed to reside just above the crypt base—around the so-called +4 position—based on DNA-label retention [11] and lineage tracing with eGFP−IRES−Cre^ER^ reporters inserted into the loci encoding BMI1 [12], mTERT [13], HOPX [14], or LRIG1 [15]. These slow-cycling reserve populations can be mobilized post injury to replenish lost or damaged *Lgr5*^+^ ISCs [9,12,13,14,15,16,17,18,19]. In addition, multiple lineage-committed progenitors and fully differentiated cell types can dedifferentiate and regain stem-like traits. Notably, *Alpi*^+^ enterocyte precursors [20], *Prox1*^+^ enteroendocrine-lineage cells co-expressing tuft-cell markers [21] and other subsets of enteroendocrine cells [22,23,24], CD69^+^CD274^+^ goblet cell precursors [25], secretory progenitors expressing *Dll1* [26] or *Atoh1* (also known as *Math1*) [27,28,29,30], differentiated KRT20^+^ surface enterocytes in the colon [31], as well as post-mitotic tuft [32], enterochromaffin [33], and Paneth cells [34,35,36] can contribute to varying degrees to crypt homeostasis and injury-induced regeneration (Figure 2b).

Classification of ISCs is further confounded by the expression of markers of +4/reserve ISCs (*Bmi1*, *mTert*, *Hopx*, *Lrig1*) in *Lgr5*^+^ cells, suggesting considerable overlap between these populations [37,38,39]. Indeed, *Lgr5*^+^ and +4/reserve ISCs can dynamically interconvert during both homeostatic and radiation-induced regeneration, although +4/reserve ISCs contribute only weakly to daily turnover under non-pathological conditions [9,14,16,17]. Nevertheless, most +4/reserve ISC populations (including *Dll*^+^ secretory precursors) differ profoundly from *Lgr5*^+^ ISCs as they are relatively refractory to Wnt stimulation, lack expression of canonical Wnt-target genes, and exhibit resistance to high-dose irradiation [14,16,17,40,41].

Recent insights have also highlighted heterogeneity within the *Lgr5*^+^ compartment itself. Addressing a long-standing controversy in the field, Buczacki and colleagues identified label-retaining cells as *Lgr5*^+^eGFP^hi^ secretory precursors of Paneth and enteroendocrine cells that do not contribute to homeostasis [30] and are discrete from the +4/reserve ISCs marked by Cre^ER^ knock-in reporters [42]. Two additional slow-cycling *Lgr5*^+^ ISC subpopulations, expressing *Mex3a* [43] or *Krt15* [44], were found to survive genotoxic stress and contribute to radiation-induced regeneration. In this respect, these slow-cycling *Lgr5*^+^ ISC subsets exhibit purported traits of +4/reserve ISCs and markedly contrast with the highly proliferative, radio-sensitive *Lgr5*^+^ ISC population. Furthermore, a rapidly-cycling, DNA damage-resistant subpopulation of *Msi1*^+^ cells, that expresses little-to-no *Lgr5* and resides at the +4 position, has recently been shown to repopulate the intestinal epithelium post irradiation [45]. Crucially, *Msi1*^+^ cells are mobilized before the reappearance of *Lgr5*^+^ cells, challenging the widely held contention that +4/reserve ISCs must regain *Lgr5* expression prior to instigating repair [46]. Although able to repopulate all major intestinal lineages, *Msi1*^+^ cells preferentially differentiate into Paneth cells, suggesting that they may first replenish the ISC niche to help restore *Lgr5*^+^ ISC functionality in the newly remodelled crypt [45]. An additional distinct—but transient—population comprises the immediate progeny of *Lgr5*^+^ ISCs, expressing modestly reduced levels of ISC-associated transcripts alongside markers of mature secretory cells and enterocytes [47]. An example of multilineage gene priming, this transient bipotential progenitor population is poised to lose *Lgr5* expression entirely as cells move further from the crypt base along their ultimate cell-fate trajectory [47,48]. Collectively, these findings suggest considerable overlap and dynamic interconversions between crypt ISC populations and implicate the local niche as the main “influencer” of stem-like behavioural and phenotypic traits.

Bringing a long-standing debate to an apparent close [49], recent studies have attributed the bulk of intestinal epithelial regeneration to the dedifferentiation of recent progeny of *Lgr5*^+^ ISCs. Both absorptive and secretory cell lineages are recruited to replenish the stem-cell pool post injury, with the underlying kinetics precluding the mobilization and expansion of dedicated reserve ISC populations [50]. Mechanistically, such pervasive dedifferentiation is underpinned by a permissive open chromatin configuration in progenitor cells undergoing differentiation [51], with only incremental chromatin remodelling of lineage-restricted genes required to interconvert between homeostatic *Lgr5*^+^ ISCs and their secretory and absorptive eventual progeny during differentiation, and vice versa during crypt regeneration [25,51].

While at times confounding, these studies collectively converge on the fact that most, if not all, crypt-resident cell types display phenomenal plasticity and retain (dormant) stemness potential, calling into question the existence of a “dedicated” reserve ISC pool. Importantly, they underscore the notion that stemness is not a cell-intrinsic trait and refocus attention on the role of the niche in governing ISC function during homeostasis and the de novo acquisition of stemness in times of stress.

## 3. Principal Niche-Signalling Pathways

**Figure 3:**
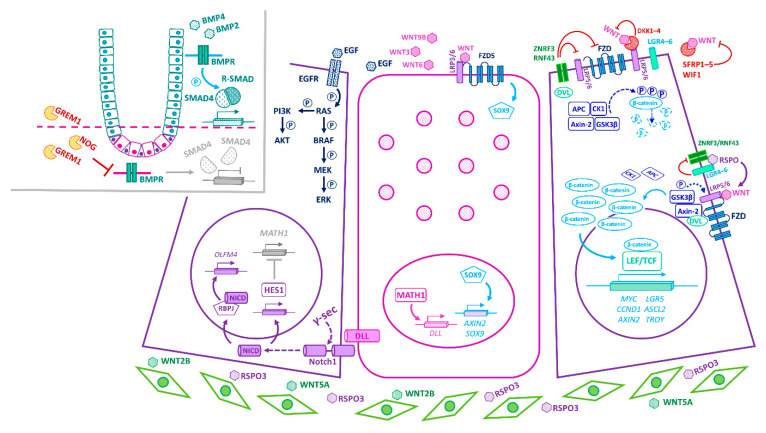
Principal niche-signalling pathways in small-intestinal homeostasis. The schematic represents *LGR5*^+^ ISCs within the niche at the crypt base, flanked by Paneth cells (centre) and pericryptal fibroblasts. Key components of the Wnt, Notch, BMP, and EGF pathways are indicated. Paneth cells present membrane-bound Notch ligands (DLLs) and secrete WNT3, WNT6, WNT9B, and EGF, as well as antimicrobials and lactate (not shown). Multiple pericryptal fibroblast populations differentially secrete agonists and antagonists of key niche pathways along the vertical crypt axis. *Upper right*: In the absence of RSPO-binding, the WNT-receptors FZDs (FZD1–10) are targeted for degradation by the E3-ubiquitin ligases RNF43 and ZNRF3. Furthermore, the Wnt antagonists SFRPs (SFRP1–5) and WIF1 sequester secreted WNT ligands, whereas DKKs (DKK1–4) bind to the LRP5/6 receptors, preventing WNT-ligand binding. Consequently, cytoplasmic β-catenin is bound by the scaffolding proteins APC and Axin-2, and sequentially phosphorylated by the kinases CK1 and GSK3β, marking it for proteasomal degradation. *Lower right*: RSPOs (RSPO1–4) bind the LGR-family of receptors (LGR4–6) and potentiate canonical Wnt signalling by inhibiting the degradation of FZDs by RNF43 and ZNRF3. Upon binding of WNT ligands to their cognate FZD and LRP co-receptors, DVL and Axin-2 are recruited to the membrane, triggering the phosphorylation of LRP5/6 by GSK3β. This, in turn, culminates in the dissociation of the destruction complex, leading to stabilization of β-catenin and its translocation to the nucleus, where it drives LEF/TCF-dependent transactivation of Wnt-target genes. *Lower left and middle*: Binding of Notch ligands (DLL1, DLL4), on the surface of Paneth cells, to juxtaposed Notch receptors (NOTCH1, NOTCH2) of adjacent *LGR5*^+^ ISCs triggers the proteolytic release of NICD, resulting in Notch-pathway activation in *LGR5*^+^ ISCs and its suppression in Paneth cells. As a result, the transcriptional repressor HES1 is activated in *LGR5*^+^ ISCs and ISC-associated markers are transcribed. In Paneth cells, the transcription factor MATH1 enhances *DLL* expression, and WNT/FZD5 transduction drives SOX9-dependent differentiation and expression of Wnt-target genes (*AXIN2*, *SOX9*) but not stemness genes (*ASCL2*, *LGR5*). *Upper left*: Binding of EGF to EGFR activates multiple downstream signalling cascades, including the RAS/BRAF/MEK/ERK and PI3K/AKT pathways which promote proliferation and survival, respectively. *Inset*: BMP activity forms a decreasing gradient from the intestinal lumen to the crypt base. Near the lumen, BMP signal transduction is initiated upon the binding of BMP ligands to their cognate BMPR receptors, leading to phosphorylation of R-SMADs, formation and nuclear translocation of R-SMAD/SMAD4 complexes, and transactivation of target genes involved in differentiation, cell-cycle withdrawal, and apoptosis. At the crypt base, pericryptal stromal cells secrete BMP antagonists (GREM1, CHDL1), protecting ISCs and progenitors from the cytostatic effects of BMPs. Infiltrating immune cells, endothelial cells, enteric neurocytes, and ECM components that also contribute to the niche are not shown. Solid arrows indicate direct activation, dashed arrows signify multiple intermediary steps, lines ending with a bar denote inhibition, grey colouring indicates suppression/inactivation, and circled P indicates phosphorylation.

Isolated *Lgr5*^+^ ISCs cultured in Matrigel supplemented with mesenchymal-derived growth factors—namely, the mitogen EGF, the Wnt agonist RSPO, and the BMP inhibitor NOG—can generate organoids that recapitulate the polarity, organization, and cellularity of the crypt–villus configuration [8]. Nevertheless, co-cultured Paneth cells significantly augment organoid-forming efficiency [52], hinting at a more complex interplay of epithelial and mesenchymal components in the niche in vivo. Indeed, the balance between ISC self-renewal and differentiation in vivo is tightly regulated through the integration of multiple epithelial- and stromal-derived cues, with primary signalling inputs from the Wnt, Notch, and BMP pathways (Figure 1 and Figure 3).

Wnt signalling has long been implicated in the maintenance of intestinal homeostasis and ISC function. In the absence of Wnt signalling, cytoplasmic β-catenin is targeted for degradation by the APC-dependent destruction complex. Binding of WNT ligands to their cognate FZD and LRP co-receptors dissociates the destruction complex and promotes nuclear translocation of β-catenin, driving LEF/TCF-dependent transactivation of Wnt-target genes (e.g., *Myc*, *Ccnd1*) (Figure 3). Genetic deletion of various Wnt-pathway components results in crypt ablation, ISC differentiation, and depletion of secretory lineages [53,54,55,56,57,58], underscoring the crucial role of this pathway in intestinal homeostasis. Conversely, APC-deficient *Lgr5*^+^ ISCs drive rapid adenoma formation in the mouse small intestine [59]. Indeed, aberrant Wnt signalling underpins colorectal cancer (CRC) initiation and progression in humans, with loss-of-function mutations in the *APC* tumour suppressor gene reported in >80% of sporadic cases and germline mutations predisposing to familial adenomatous polyposis [60]. Notably, tumorigenesis selects for *APC* mutants with a residual ability to downregulate β-catenin [61] and, furthermore, the spectrum of *APC* mutations in tumours varies along the length of the intestine, reflecting local variations in Wnt-signal strength [62]. Thus, a “just-right” level of Wnt activity sustains growth of early dysplastic lesions [61] and promotes mutant fixation [63], whereas over-activation of Wnt signalling elicits apoptosis and counters polyp formation [61].

RSPOs (RSPO1–4) bind the LGR family of receptors (LGR4–6) and potentiate canonical Wnt signalling by inhibiting the degradation of the WNT-receptors, FZDs (FZD1–10), by the E3-ubiquitin ligases RNF43 and ZNRF3 [4,6,64,65]. While WNTs and RSPOs synergize to augment Wnt signalling, they each serve distinct roles within the niche. Notably, WNT ligands alone cannot evoke *Lgr5*^+^ ISC self-renewal; instead, they maintain basal RSPO-receptor (*Lgr5*, *Rnf43*, *Znrf3*) expression, priming *Lgr5*^+^ ISCs for RSPO-driven proliferative expansion. Thus, RSPOs maintain *Lgr5*^+^ ISCs in a multipotent, undifferentiated state and, crucially, control the size of the *Lgr5*^+^ ISC pool in a Wnt-dependent manner [66]. Independently of LGR5, however, a recent study identified an important role for stromal RSPO3 in orchestrating the colonic wound-healing response to treatment with dextran sodium sulphate (DSS), a widely used chemical colitogen that acutely damages the colonic mucosa [67,68,69]. Here, RSPO3 reprograms *Lgr5*^−^*Lgr4*^+^ differentiated cells (by engaging the RSPO3-receptor LGR4) into a Wnt-high, stem-like state, capable of supporting epithelial regeneration following DSS-induced *Lgr5^+^* ISC depletion [31]. These data suggest that a rewired niche interactome may instruct cell fate during tissue distress.

In homeostasis, canonical Wnt activity prevails at the crypt base and the associated target-gene expression declines as cells ascend the vertical crypt axis [70]. In addition to *Lgr5*—itself a Wnt-target gene—*Lgr5*^+^ ISCs express other Wnt targets, including *Ascl2*, *Sox9*, *Troy*, and *Axin2* [39,71,72], underscoring the importance of Wnt signalling for safeguarding the stem cell state at the crypt base [72]. ASCL2—a master regulator of the *Lgr5*^+^ ISC gene expression program—perpetuates its own expression in a positive feedback loop, controlled by WNT/RSPO levels. Thus, while an “*Ascl2*-on” state imparts stemness, “*Ascl2*-off” cells are destined to differentiate, with the corollary that TA cells can regain stemness upon encountering increased WNT/RSPO levels sufficient to drive *Ascl2* expression [73]. In fact, the induction of *Ascl2* is critical for the ability of recent *Lgr5*^+^ ISC progeny to undergo dedifferentiation to an *Lgr5*^+^ state prior to regenerating the injured intestinal epithelium [50].

The Notch pathway also regulates the proliferation and multipotency of *Lgr5*^+^ ISCs, while additionally specifying TA cell fate [74,75,76]. Briefly, binding of transmembrane Notch ligands (DLL1, DLL4, JAG1, JAG2) to juxtaposed Notch receptors (NOTCH1, NOTCH2) of adjacent cells results in Notch-pathway suppression in the signal-sending cell and activation in the receiving cell, a process known as lateral inhibition [77]. The NOTCH receptor is proteolytically cleaved by γ-secretase, liberating its transcriptionally active intracellular domain (NICD), which converges with the coactivator RBPJ to drive Notch target-gene expression (Figure 3). In the TA-cell compartment, abrogation of Notch signalling enforces differentiation of proliferative cells into secretory cells via recruitment of MATH1, the master regulator of secretory fate [74,78,79]. Conversely, constitutive Notch signalling activates the transcriptional repressor, HES1, prompting a switch to an enterocyte fate [75,76,80,81].

The constitutively activated Notch phenotype is also notable for the marked expansion of crypt progenitors, implicating Notch signalling in this compartment [75]. Consistent with this notion, the NOTCH1 and NOTCH2 receptors are restricted to *Lgr5*^+^ ISCs, whereas the instructive Notch ligands DLL1 and DLL4 are expressed on Paneth cells in the small intestine [52,82] or DCS cells in the colon [3]. Moreover, activity of the Notch-effector HES1 is detected in *Lgr5*^+^ ISCs as well as absorptive progenitors [52,83]. Accordingly, simultaneous deletion of *Dll1* and *Dll4*, or pharmacological inhibition of Notch, elicits the conversion of proliferative *Lgr5*^+^ ISCs into post-mitotic goblet cells, concomitant with loss of ISC-associated markers (e.g., *Lgr5*, *Olfm4*, and *Ascl2*) [82,84]. Conversely, constitutive activation of Notch signalling in Paneth cells prompts their dedifferentiation into a stem-like *Lgr5*^+^ state [35,36]. These findings underscore the requirement of Notch signalling for the survival, maintenance, and activity of the *Lgr5*^+^ ISC pool as well as for cell-fate decisions. Moreover, whereas secretory lineage commitment requires MATH1, transcription of the ISC marker and Notch target, *Olfm4*, requires RBPJ and occurs independently of MATH1 [84], implicating distinct downstream Notch-effector pathways in cell-fate specification and ISC maintenance.

BMP activity counters Wnt signalling, forming a decreasing gradient from the intestinal lumen to the crypt base [70,85] (Figure 1 and Figure 3). BMP signal transduction is initiated upon binding of BMP ligands to their cognate BMP-receptors (BMPRs), leading to phosphorylation of R-SMADs (SMAD1, SMAD5, SMAD8), formation and nuclear translocation of R-SMAD/SMAD4 complexes, and transactivation of target genes [86]. BMP ligands (BMP2, BMP4) abound near the lumen, promoting cell-cycle withdrawal, differentiation, and apoptosis of luminal epithelial cells [85,87,88]. Conversely, at the crypt base, pericryptal stromal cells secrete the BMP antagonists GREM1, GREM2, and NOG, which sequester the BMP ligands away from their cognate receptors, thereby protecting ISCs and progenitors from the cytostatic effects of BMPs [70,88,89,90,91,92]. Accordingly, acute *Grem1* deletion precipitates a severe enteropathy with profound tissue atrophy, consistent with the demise of proliferative *Lgr5*^+^ ISCs [93]. On the other hand, aberrant expression of *Grem1* [94] or *Nog* [87] in the mouse intestinal epithelium severely disrupts BMP-morphogen gradients, leading to the formation of ectopic crypts and polyps with a pathology reminiscent of human polyposis syndromes [87,94,95], typically associated with mutations in BMP-pathway genes and a high-risk predisposition to CRC [96]. Similarly, simultaneous deletion of *Bmpr1a* in the stromal and epithelial compartments yields hyperproliferative crypts that eventually lead to polyposis [88]. These phenotypes are underpinned by the marked expansion of the stem/progenitor cell compartment, consistent with a homeostatic role for BMP antagonists in restricting the self-renewal of *Lgr5*^+^ ISCs at the crypt base [87,88,94,95]. Notably, however, epithelial-specific deletion of *Bmpr1a* does not elicit de novo crypt formation, thereby selectively implicating the loss of stromal BMP signalling in the pathology of polyposis [97]. Instead, these mice exhibit increased crypt fission as well as impaired goblet, Paneth, and enteroendocrine cell maturation, linking epithelial BMP signalling to secretory cell fate [97]. Mechanistically, epithelial BMP signalling selectively drives SMAD/HDAC1-mediated repression of stem cell-associated genes (e.g., *Lgr5*, *Sox9*, *Pdgfa*, *Cdk6*, and *Cdca7*)—notably without impacting β-catenin nuclear translocation or expression of non-stem canonical Wnt targets (e.g., *Ccnd2*, *Axin2*, and *Myc*)—thus safeguarding against precocious *Lgr5*^+^ ISC expansion and polyp formation in the Wnt-rich niche [95]. Overall, BMP signalling maintains homeostatic balance by dampening the proliferation of *Lgr5*^+^ ISCs at the crypt base while promoting secretory cell lineage allocation in luminal regions.

Thus, within the confines of the crypt base, *Lgr5*^+^ ISCs are nurtured with convergent niche signals that preserve their self-renewal capabilities and multipotency. But no one signalling pathway acts in isolation. Below, we touch on the crosstalk between key niche-signalling pathways and delineate how this shapes an instructive milieu that supports stemness and dictates cell fate.

## 4. Convergence of Niche-Signalling Pathways in Homeostasis

The Wnt and Notch pathways function synergistically to preserve the proliferative activity and multipotency of *Lgr5^+^* ISCs. According to the emergent model, DLL1^+^DLL4^+^ Paneth cells activate Notch signalling in adjacent *Lgr5*^+^ ISCs at the crypt base, maintaining them in an undifferentiated state by tempering Wnt signalling [98] and suppressing secretory lineage specification [82,99]. Additionally, opposing inputs from the Wnt and BMP pathways control expression of stem-cell-associated signature genes and, consequently, ISC renewal [70,90,92,95]. EGF is also a major driver of *Lgr5*^+^ ISC proliferation, but it is not linked to stemness potential per se [100]. In fact, Wnt suppresses EGFR/MAPK activity in vivo to maintain quiescent ISC pools and prevent their premature differentiation into progenitors [101]. Furthermore, *Lgr5*^+^ ISCs themselves secrete the Notch target OLFM4—an inhibitor of proliferation, inflammation, and Wnt/β-catenin signalling [72,102,103]—and the BMP inhibitor SMOC2 [39], which in turn likely modulate the niche-signalling output.

Of note, several studies have detected transcripts encoding Wnt antagonists, such as *Axin2*, *Sfrp1*, *Sfrp2*, *Sfrp5*, *Fzdb*, *Dkk2*, *Dkk3*, *Wif1*, and *Notum*, differentially localized in crypt epithelial cells or the adjacent mesenchyme [92,104,105,106]. Whereas these molecules often have different modes of action and variegated expression patterns, the raison d’être of so many different Wnt antagonists near the crypt base remains unclear. Their presence, indeed, points to the need to constantly fine-tune homeostatic Wnt-pathway activity and ISC function, with the corollary that the breakdown of such negative feedback loops will likely bear relevance to the emergence of disease. For example, with advancing age, Paneth cells increasingly secrete NOTUM—a WNT deacylase that disrupts WNT-ligand binding to FZD receptors [107]—thus limiting the capacity of neighbouring ISCs to self-renew and regenerate the intestinal epithelium, and compromising intestinal function over the longer term [106]. Currently in preclinical development, NOTUM inhibitors may therefore find therapeutic utility in pathologies associated with attenuation of Wnt signalling. For example, they may be employed to help restore the regenerative capacity of the aging intestinal epithelium or to ameliorate the ravages of chemotherapy and irradiation [108]. Deciphering the precise target-cell populations of Wnt antagonists deployed within the niche, how their various modes of action converge or diverge at the crypt base, and how their functional outputs are altered in regeneration and disease, will better inform our understanding of crypt dynamics and how to right the balance between self-renewal and differentiation when gone awry.

Multiple different signals converge to specify lineage choice as cells exit the stem cell compartment to ascend the crypt. In the TA zone, Notch activity confers an absorptive cell fate, whereas Delta/Notch lateral inhibition allows secretory commitment. Having lost contact with Paneth cells, displaced ISCs stochastically begin to express *Math1*, committing to a secretory fate as DLL1^+^DLL4^+^ progenitors, which subsequently provide instructive Notch signals to ascending neighbours, fating them toward an enterocyte lineage [26,99]. Concurrent inhibition of Notch, Wnt, and EGFR signalling—which respectively control enterocyte, Paneth, and goblet cell fates—converts *Lgr5*^+^ ISCs into multiple enteroendocrine subtypes [74,100]. BMP signalling also cooperates with local signals to promote terminal differentiation of secretory lineages [97].

In the absence of Notch signalling [109], WNT/FZD5 transduction drives SOX9-dependent maturation of Paneth cells [110,111,112,113]. Indeed, mature Paneth cells exhibit active Wnt/β-catenin signalling and express Wnt-pathway genes (e.g., *Axin2*, *Sox9*, *Wnt3*) but, notably, lack expression of stemness genes (e.g., *Ascl2*, *Lgr5*) [41]. Moreover, Wnt/EphB-signalling crosstalk ensures correct positioning of Paneth cells at the crypt base [114]. These findings suggest differential deployment of distinct Wnt-transduction cascades, within the crypt base, to confer stemness or specify Paneth cell fate. Interestingly, while dramatic loss of *Lgr5*^+^ ISCs upon DSS-driven acute inflammation promotes dedifferentiation and cell-cycle re-entry of Paneth cells (or their lineage-committed precursors) via the SCF/cKit/Wnt-signalling axis [34], DNA damage engages the Notch pathway [35,36]. These studies suggest that different niche effectors may drive regeneration depending on the mode and severity of injury [34,35,36].

In sum, the output of Wnt signalling is fine-tuned by localized concentration gradients of RSPOs, LGR and FZD receptors [109], Wnt antagonists [104], and components of the Notch [98], BMP [70,87,88,90], and EGF [100,101] pathways. Below, we discuss the niche constituent cell types that elaborate these signals.

## 5. Cellular Circuitries

Paneth cells provide WNT3, WNT6, WNT9B, EGF, Notch ligands, antimicrobials, and lactate, which sustain the proliferative and metabolic activity of *Lgr5*^+^ ISCs [52,104,109,115]. REG4^+^ DCS cells—purported to be the colonic equivalent of Paneth cells—provide EGF and the Notch ligands DLL1 and DLL4, enhancing survival of colonic *Lgr5*^+^ ISCs in vivo and in vitro [3,116]. Notably, DCS cells do not secrete canonical WNTs, suggesting the existence of alternative sources of WNTs in colonic crypts and, likely, accounting for the dependency of cultured colonic organoids on exogenous WNT3A [117].

Surprisingly, epithelial-specific deletion of *Wnt3* [109] or inhibition/deletion of PORCN (an acyltransferase essential for WNT secretion) [118,119], or Paneth cell ablation [120,121,122] does not perturb ISC maintenance, intestinal homeostasis, or radiation-induced regeneration. These studies, therefore, concur that epithelial-derived WNTs are redundant in these settings in vivo. Taken together with the fact that the simultaneous deletion of *Wls* in both the epithelial and mesenchymal lineages abrogates WNT secretion and severely impairs homeostasis [58], these findings contend that crypt-associated stromal cells likely comprise an important physiological source of Wnt ligands. By contrast, epithelial-derived WNTs have been ascribed an essential role in mobilizing unharmed *Lgr5*^+^ ISCs (but not +4/reserve ISCs) to replenish differentiated villus epithelial cells, damaged by rotavirus infection [123]. In this instance, epithelial-specific deletion of the WNT-ligand secretion mediator, *WIs*, thwarted the repair process, implicating secreted epithelial-derived WNT ligands in the recruitment of *Lgr5*^+^ ISCs post rotavirus infection [123]. In addition, the small intestinal epithelium can serve as a compensatory WNT source when WNT secretion from *Gli1*^+^ mesenchymal cells is hindered [124].

Whereas deletion of *Porcn* in subepithelial myofibroblasts failed to elicit a discernible phenotype [119], various potentially overlapping pericryptal fibroblast populations, expressing *Foxl1* [89,105], *Pdgfrα* [125], *Gli1* [58,124], or CD34^+^Gp38^+^ [91], have been shown to furnish the niche with abundant WNT ligands. Perturbation of these mesenchymal cell types (by lineage ablation, or deletion of *Porcn* or *Wls*) drastically impaired crypt proliferation in homeostatic and/or pathological settings [58,89,91,124,125]. All these stromal populations express high levels of WNT2B [58,105], which can rescue *Wnt3*^−/−^ organoids from death [109]. Moreover, like WNT3, WNT2B can bind FZD7, a critical receptor for crypt homeostasis and regeneration [57].

Recent technical advances have illuminated and refined our understanding of how distinct sub-epithelial stromal populations generate the opposing gradients of Wnt- and BMP-pathway activity along the crypt–villus axis in the small intestine. Various subpopulations of telocytes—*Foxl1*^+^PDGFRα^+^ subepithelial myofibroblasts so-called after their long cytoplasmic protrusions (telopodes) that envelop crypt cells—differentially express genes encoding secreted agonists and antagonists of key niche pathways, including WNT2B, WNT5A, RSPO3, the Wnt inhibitors DKK3 and SFRP1, various BMPs (BMP4, BMP5, BMP6, and BMP7), and the BMP inhibitors CHRDL1 and GREM1, providing local positional cues [105]. For example, *Foxl1*^+^PDGFRα^+^ telocytes express higher levels of *Wnt2b* and *Rspo3* at the crypt base, stimulating Wnt signalling in apposing *Lgr5*^+^ ISCs. Conversely, transcripts encoding the non-canonical ligand WNT5A, the Wnt antagonists sFRP1 and DKK3, and BMP5 are enriched near the crypt–villus junction, relaying local differentiation cues [105].

PDGFRα^+^ mesenchymal populations have further been subclassified into two distinctly localized pools, tasked with establishing the diminishing BMP gradient from the top to the crypt bottom. First, PDGFRα^hi^ telocytes that predominate at the crypt–villus junction, as well as the villus tip, supply diffusible BMP agonists luminally, driving local cell differentiation [92]. Interestingly, PDGFRα^hi^ telocytes are the only gut-resident stromal cell known to express BMP7, which is noteworthy given that BMP2/BMP7 and BMP4/BMP7 heterodimers are significantly more potent signal transducers than their respective homodimers [92,126]. Second, newly identified CD81^+^PDGFRα^lo^ subcryptal trophocytes—named for their ability to support ISC expansion in vitro in the absence of exogenous factors (i.e., Wnts, RSPOs, and BMP inhibitors)—release the BMP inhibitor GREM1 at the crypt base, safeguarding the self-renewal capacity of ISCs against diffusible BMP ligands. These subcryptal trophocytes also secrete RSPO1 and RSPO2 to augment local Wnt signalling [92]. Dotted among smooth-muscle fibres and running parallel to the crypt–villus axis, an abundant third population of CD81^−^PDGFRα^lo^ interstitial fibroblast-like cells is enriched for *Cd34*, *Pdpn* and *Gli1* transcripts, and can secrete RSPO1, but its functional contribution to the niche remains unknown [92].

Intriguingly, a recent study reported the surprising observation that a novel sub-population of PDGFRα^+^ telocytes, localized at the villus tip, expresses the crypt stem-cell marker *Lgr5* as well as *Rspo3* [127]. Ablation of these *Lgr5*^+^ villus-tip telocytes, which also express *Bmp4*, *Wnt4*, *Wnt5A*, and *Egf*, profoundly altered the gene-expression profiles of apposing enterocytes, approaching the villus tip prior to sloughing off into the lumen. While these results have been met with some scepticism [1,128], any validation of *Lgr5*^+^ villus-tip telocytes as a *bona fide* stromal cell could have far-reaching implications for approaches relying on *Lgr5*^+^ driven Cre-mediated gene targeting or diphtheria toxin-mediated ablation of cells engineered to express the diphtheria-toxin-receptor, DTR, under the control of the *Lgr5* promoter (*Lgr5^DTR^*) [127]. Further studies should interrogate which *Lgr5* transcript variants are expressed in villus-tip telocytes and probe the significance of their distinctive compartmentalization within telopodes [127]. In addition, such follow-up studies will need to establish the fidelity of lineage tracing and cell ablation in this model, given that villus-tip telocytes have been “dismissed” as subcryptal trophocytes [128], and others have pointed out that tuft cells may also be depleted with this strategy [1].

Collectively, the above studies demonstrate that multiple different cellular circuitries converge to provide essential—but at times redundant—morphogens to support homeostatic turnover of the intestinal epithelium.

## 6. The Immune Niche

Even in homeostatic conditions the intestine is under constant inflammatory assault from its high microbial load and the influx of dietary antigens. Multiple heterogeneous populations of long-lived macrophages, residing within discrete niches along the intestinal tract, differentially display bactericidal activity, help mop up apoptotic debris (efferocytosis), and deploy during wound healing to ensure barrier integrity [129]. In addition to their roles in immune surveillance and host defence, both gut-resident and tissue-infiltrating immune cell types have been increasingly recognized as important contributors to the ISC niche. Notably, antibody-mediated blockade of CSF1R-dependent crypt-associated macrophages perturbed Paneth cell differentiation, thereby leading to depletion of *Lgr5*^+^ ISCs, functional impairment of M cells, and a shift toward goblet cell differentiation [130]. The effects of CSF1R blockade on crypt homeostasis are likely to be two-fold: indirect by perturbing the differentiation of Paneth cells and direct by depriving *Lgr5*^+^ ISCs of macrophage-produced *Wnt4* and *Rspo1* [130]. Additionally, post irradiation, gut-resident macrophages can secrete extracellular vesicles containing WNT5A, WNT6, and WNT9A, thereby supporting the ensuing regenerative response and augmenting *Lgr5^+^* ISC mobilization [131]. Thus, gut-resident macrophages support crypt homeostasis in the small intestine and promote mucosal repair post damage, with the corollary that immunogenic challenge could adversely impact the balance of differentiation in the intestinal epithelium and, by extension, its functions in immune surveillance. Interestingly, prolonged CSF1R blockade led to the expansion of SOX9^+^*Bmi1*^+^ cells, demonstrating the mobilization of an *Lgr5*^−^ reserve ISC pool that nevertheless failed to restore the proportions of epithelial cell lineages in the villus [130].

Aside from macrophages [130,131], innate lymphoid cells have also been shown to directly influence *Lgr5*^+^ ISC function [132,133]. For example, group 3 innate lymphoid cells and γδ T cells secrete IL22, which induces STAT3 phosphorylation in *Lgr5*^+^ ISCs, driving organoid growth and epithelial regeneration post damage, independently of the Paneth cell niche [132]. Furthermore, in response to AhR-mediated signalling, IL22 can engage the DNA-damage response machinery, protecting *Lgr5*^+^ ISCs from genotoxic dietary compounds and the acquisition of deleterious mutations [134]. Thus, multiple innate immune cell types, and the cytokines they produce, play pleiotropic roles within the ISC niche that go beyond host defence against pathogens to impact ISC fate and differentiation trajectory.

Adaptive immune T cells have also recently emerged as an important determinant of the ever-pliant immunological milieu [135]. In a landmark study employing single-cell RNA-sequencing, Biton and colleagues identified two distinct small-intestinal *Lgr5*^+^ ISC subsets (ISC-II and ISC-III) that express components of the major histocompatibility complex class II (MHC-II) machinery. These proliferative, relatively differentiated *Lgr5*^+^ ISC subsets can present processed antigens to naive T-helper (Th) cells and induce T-cell activation or tolerance, consequently serving as non-conventional antigen-presenting cells [135]. By contrast, a third more quiescent ISC-I subset displays minimal MHC-II expression and is endowed with a more stem-like gene expression signature. Conditional ablation of MHC-II components in intestinal epithelial cells enriched for *Lgr5*-expressing cells in crypt regions and decreased CD4^+^ T cells in the crypt lamina propria, strongly implicating peptide-MHC-II interactions in the regulation of the balance of cell types in the vicinity of the crypt. Reciprocally, activated Th cells produce distinct cytokines that influence the balance between self-renewal and differentiation of *Lgr5^+^* ISCs [135]. In organoid co-cultures, proinflammatory signals, such as the presence of Th1, Th2, or Th17 cells or treatment with IL13 or IL17, promoted differentiation, enriching for TA cells with a concomitant reduction in ISCs [135]. In fact, each Th subset impacted the ISC differentiation trajectory differently: Th1 co-cultured organoids were enriched for the Paneth and goblet cell lineages, Th2 signals induced an enteroendocrine phenotype, Th17 cells or their cytokine IL17a reduced ISC renewal and promoted a TA cell fate, and IL13 treatment favoured tuft cell differentiation over Paneth and goblet cell types [135].

Regulatory T cells (Tregs)—a subset of CD4^+^ helper T lymphocytes that curtail inflammatory immune responses to avert the development of detrimental autoimmunity—additionally regulate intestinal homeostasis by directly supporting ISC self-renewal [135,136]. Accordingly, co-culture of intestinal organoids with the anti-inflammatory Tregs, or their secreted cytokine IL10, led to expansion of the *Lgr5*^+^ ISC pool [135]. Conversely, mice with Treg ablation exhibited a reduction in the ISC-I subset and a shift toward the more proliferative MHC-II^+^ *Lgr5*^+^ ISC-II and ISC-III subsets, consistent with depletion of ISC-I cells through increased differentiation and coincident with the recruitment of pro-differentiative Th (Th1, Th2, and Th17) cells to crypt regions [135].

The crosstalk between immune cells and *Lgr5*^+^ ISCs also helps shape the small-intestinal niche post infection. Here, the balance is skewed toward the more differentiated MHC-II^+^ *Lgr5*^+^ ISC-II and ISC-III subsets with concomitant suppression of the ISC-I state [135]. Post bacterial infection, Th1 cytokines promote differentiation toward the Paneth cell lineage, consistent with a requirement for the antimicrobial peptides that these cells secrete into the niche [135,137]. On the other hand, helminth and protist infections mobilize tuft cells to secrete IL25, leading to release of IL13 and IL4 from group 2 innate lymphoid cells [133,138,139]. IL13, in turn, acts on epithelial crypt progenitors to evoke differentiation of tuft and goblet cells, amplifying the so-called “weep and sweep” response whereby increased mucus (weep) and muscle contractility (sweep) expel the helminth parasite from the intestinal lumen [133,139]. Interestingly, these parasites trigger a profound remodelling and lengthening of the small intestine, underpinned by tuft cell hyperplasia, which serves to perturb further parasite colonization—a phenomenon termed concomitant immunity [140]. Importantly, deletion of MHC-II in *Lgr5*^+^ ISCs compromised the tuft cell mobilization seen in control infected counterparts and increased overall helminth load [135]. Together, these findings underscore the importance of the crosstalk between *Lgr5*^+^ ISCs and innate and adaptive immune cells in determining the balance between stemness and differentiation both in homeostasis and post inflammatory insult [135]. Below, we further delineate the cellular and molecular circuitries that shape the niche post injury.

## 7. Niche Remodelling Post Injury

Although most studies have delineated the different crypt cell types that can mobilize to replenish *Lgr5*^+^ ISCs post injury, the niche itself can also remodel and adapt to damage. Thus, following Paneth cell ablation, enteroendocrine and tuft cells can be recruited to the crypt base as a reserve source of instructive Notch signals, enabling the maintenance and proliferation of *Lgr5*^+^ ISCs to continue unabated [122]. Another example of niche remodelling is observed following acute, transient Notch inhibition. Somewhat surprisingly, this triggers rapid apoptotic demise of Notch ligand-bearing Paneth cells, leaving *Lgr5*^+^ ISCs intact, albeit with diminished lineage-tracing capacity. Nevertheless, in this setting, both *Lgr5*^+^ ISCs (that activate expression of *Dll1*) and *Dll1*^+^ multipotent progenitors can mobilize to replenish the depleted Paneth cell pool and restore Notch homeostasis [141]. While these results contrast with the loss of *Lgr5*^+^ ISCs and the expansion of Paneth-like cells observed during prolonged Notch inhibition [84], they attest to the potential tolerability of transient Notch perturbation in the clinic and underscore that different modes of injury elicit distinct cellular and molecular responses.

Epithelial damage also leads to remodelling of the mesenchymal niche. Following DSS-induced injury, CD34^+^Gp38^+^ non-myofibroblastic pericryptal cells express several genes whose products promote stemness (e.g., *Grem1*, *Rspo1*), recruit neutrophils and macrophages to the inflamed tissue (e.g., *Il7*, *Ccl2*, *Csf1*), and facilitate epithelial restitution (e.g., *Areg*, *Fgf7*, *Fgf10*, *Col1a1*, *Ptgs2*) [91]. DSS treatment also stimulates the expression of genes encoding various BMPs, vascular remodelling factors (e.g., ANGPT1, ANGPT2, VEGFA), and WNT5A in CD34^−^ lamina propria myofibroblasts, thus promoting epithelial differentiation, repair, and regeneration of the upper villi and colonic surface epithelium [91]. In the colon, which lacks a WNT-secreting epithelial cell type, *Gli1*^+^ subepithelial mesenchymal cells serve as an essential WNT source, supporting colonic ISC renewal both during homeostasis and following DSS-induced injury [124]. As mentioned earlier, these cells can also mobilize as a reserve WNT source in the small intestine, if epithelial secretion is compromised [124].

The EGF family member NRG1 has recently been identified as an important extracellular cue that augments the proliferation of stem and progenitor cells in the regenerating epithelium through downstream activation of MAPK and PI3K/AKT signalling [142]. Following DNA damage, multiple mesenchymal populations, including CSF1R-dependent crypt-associated macrophages [130], PDGFRα^+^ subepithelial telocytes [105], and CD34^+^ PDGFRα^lo^ trophocytes [92], as well as Paneth cells secrete NRG1 (but not EGF), driving dedifferentiation of progenitor cells towards a more stem-like phenotype and promoting regeneration [142].

Whereas the intestinal epithelium displays extraordinary plasticity and can regenerate following multiple types of injury, failure to restore barrier integrity post damage risks translocation of intestinal microbiota, resulting in inflammation and likely predisposing to tumour formation. Indeed, chronic inflammation is considered a hallmark of cancer, and inflammatory bowel disease (ulcerative colitis or Crohn’s disease) is often a prelude to CRC [143]. Recent single-cell profiling of colonic tissues, from patients with ulcerative colitis and healthy donors, has illuminated the contributions of the epithelial, stromal/mesenchymal, and immune compartments to colon homeostasis and implicated their dysfunction in inflammatory bowel disease [144,145,146]. 

Until recently, little was known about the colonic mesenchyme in humans. Aside from pericytes and myofibroblasts, four distinct clusters of fibroblast-like cells have now been identified in the healthy human colon, designated stromal clusters S1–S4. Of note, the subcryptal S2 population expresses the transcription factor SOX6, the coagulation factor F3/CD142, the non-canonical WNT ligands WNT5A and WNT5B, the BMP agonists BMP2 and BMP5, the secreted Wnt antagonist FRZB, and the Th2 cytokine POSTN, consistent with a pivotal role in the paracrine control of cell proliferation and differentiation in the upper crypt [144]. 

The onset of colitis is associated with extensive mesenchymal niche remodelling. Marked depletion of the mesenchymal SOX6^+^ S2 cell population likely underlies the epithelial barrier disruption that typifies this condition. Conversely, a population of activated mesenchymal S4 cells, which is barely detectable in the normal healthy colon, expands and deploys proinflammatory and stress-response factors that recruit immune cells to the gut mucosa. These activated S4 cells express genes such as *PDPN*, typically associated with fibroblastic reticular cells (that coordinate lymphocyte migration within lymph nodes), the potent T/B-cell chemotactic factors CCL19 and CCL21, the TNF-superfamily member TNFSF14, the proinflammatory cytokine IL33, and lysyl oxidase (LOX) enzymes that remodel the ECM by cross-linking collagens and elastin [144]. Accordingly, LOX/LOXL1 blockade reduced the severity of the inflammation in a DSS-induced colitis model [144]. Overall, the demise of the S2 cluster is thought to impair the regenerative capacity of the overlying epithelium, whereas the expanded S4 cluster sustains a state of prolonged inflammation, preventing resolution of the wound-healing response and perpetuating tissue distress and barrier dysfunction [144].

Similarly, Smillie et al. identified a population of WNT2B^+^WNT5B^+^ inflammation-associated fibroblasts, uniquely expanded in the inflamed as well as the cancerous colon, and enriched for markers of colitis, fibrosis, and cancer-associated fibroblasts (CAFs) [145]. For example, one of the most highly expressed genes in this cluster, *OSMR*, encodes the receptor for oncostatin M, a cytokine known to predict resistance to the anti-TNF therapy used to treat inflammatory bowel disease [145,147]. The emergence of WNT2B^+^WNT5B^+^ inflammation-associated fibroblasts occurs alongside a marked expansion of mislocalized M-like cells, inflammatory monocytes, and CD8^+^IL17^+^ T cells, consistent with immune derailment/inflammation [145]. 

In addition to the remodelling of the mesenchymal compartment, dysfunction of epithelial-cell subsets has also been documented in patients with ulcerative colitis. Depletion of the newly identified BEST4/OTOP2 absorptive cells, implicated in pH regulation, and the emergence of malpositioned goblet cells, displaying impaired antimicrobial function, compromise the epithelial barrier and allow bacterial invasion [146]. Collectively, the above studies implicate aberrant remodelling of the epithelial, mesenchymal, and immune compartments in human ulcerative colitis, underpinned by expansion and/or depletion of newly identified rare cell types, de novo activation or repression of cell lineage-specific gene expression modules, and rewired cell-cell interaction networks—all perpetuating a highly dysfunctional inflammatory milieu.

## 8. At the Crossroads of Intestinal Regeneration and Tumorigenesis—The YAP-Driven Foetal-Like Signature

Additional studies have interrogated the molecular mechanisms underlying the response of the mouse intestinal epithelium to helminth infection [148] and DSS-induced injury [149], both of which breach the mucosal barrier. An emergent theme is that the regenerating intestinal epithelium is transiently reprogrammed into a highly plastic foetal-like state, orchestrated by changes in the inflammatory milieu [148] and ECM [149], respectively. The extensive tissue remodelling that ensues entails the deployment of highly proliferative SCA1^+^ progenitors [148,149], lacking markers of secretory lineages as well as of adult ISCs—most notably, *Lgr5* and LRIG1 [39,149]. Instead, these regenerating cells express foetal epithelial markers, such as *Anxa1* and *Tacstd2*/*Trop2*, alongside the multipotent progenitor marker SCA1 (also known as LY6A) [149,150]. Notably, although *Sca1* is absent from the human genome, *ANXA1* is highly expressed in the regenerating epithelium of inflamed ulcerative colitis, compared with non-inflamed regions in matched patient specimens [149]. Moreover, the transcriptional signatures of the mouse repairing epithelium and the foetal-like state are enriched in patients with active inflammation, compared with healthy counterparts, lending relevance of this foetal-like program to human disease [149]. Similarly, crypts overlying helminth larvae-associated granulomas become devoid of *Lgr5* expression, with a discrete subset of SCA1^+^ crypt cells activating an IFNγ-dependent foetal-like transcriptional program [148]. Indeed, similar injury-response programs are deployed following irradiation and DTR-targeted *Lgr5*^+^ ISC ablation, suggesting that the transient “revival” of latent foetal-like traits is likely a common denominator of the intestinal epithelial response regardless of the mode of injury [148].

Following DSS treatment, the regenerating mouse intestinal epithelium is also characterized by upregulation of several ECM components and the accumulation of collagen type I fibres around newly formed crypts. These dynamic changes in the ECM composition of the niche are propagated via FAK/SRC-mediated mechanotransduction, culminating in the activation and nuclear translocation of YAP and TAZ [149], two paralogous transcriptional coactivators inhibited by the Hippo tumour-suppressor pathway [151]. YAP has similarly been shown to transiently reprogram *Lgr5*^+^ ISCs into a regenerative state post irradiation. Here, YAP suppresses homeostatic Wnt signalling and Paneth cell differentiation while concomitantly activating expression of the EGF-family member EREG to drive proliferation and promote cell survival [152]. Indeed, several studies concur that YAP/TAZ can inhibit Wnt signalling during intestinal regeneration and tumorigenesis [152,153,154,155], consistent with the suppression of *Lgr5* and the ISC signature during the foetal-like regenerative response [149].

A critical role for YAP has also been ascribed in the damage-induced mobilization of “revival stem cells”, recently identified in the regenerating intestinal epithelium using a single-cell transcriptomics approach [156]. The revival stem cell pool is a rare, quiescent population in homeostasis, characterized by elevated expression of clusterin (*Clu*), *Anxa1*, *Cxadr*, and *Basp1*. While these *Clu*^+^ revival stem cells do not contribute to daily homeostatic renewal, they are mobilized and expanded following DTR-mediated ablation of *Lgr5*^+^ ISCs, irradiation, or DSS-induced inflammation and colitis. Their transient expansion post damage regenerates the full gamut of intestinal cell types, including *Lgr5*^+^ ISCs, in a YAP1-dependent manner [156]. Interestingly, *Clu*^+^ revival stem cells express elevated levels of *Sca1* post irradiation [156], raising the possibility that this damage-induced, expanded revival stem cell population overlaps with *Sca1*^+^ foetal-like crypt cells, which also rely on YAP for their regenerative potential [149].

The lipid transporter TIPE0 (also known as TNFAIP8) has recently been recognized as an important regulator of the *Clu*^+^ regenerative program, with *Tipe0^−/−^* mice exhibiting poor recovery from DSS-induced injury and reduced subsequent survival [157]. Underlying this phenotype is a broad dysfunction of plasticity programs, characterized by an overabundance of *Lgr5*^+^ ISCs and partially differentiated cells in homeostasis, and an impaired capacity to dedifferentiate post injury. Indeed, although *Tipe0^−/−^* enterocytes are induced to proliferate post injury, they fail to recruit YAP to the nucleus and are hence impeded from mounting a *Sca1*^+^*Clu*^+^ regenerative response, leading to intestinal demise [157].

Whereas the YAP-driven regenerative response is a transient, reversible process [149,156], persistent tissue injury and repair set up a vicious cycle of chronic inflammation—a known risk factor for CRC [143] as discussed previously. Indeed, the YAP-mediated regenerative response can be hijacked to facilitate the progression of APC-deficient foci to adenomas to the extent that *Yap* deletion abrogates adenoma formation in *Apc^Min/^*^+^ mice [152,158]. Moreover, the YAP transcriptional program correlates with the gene expression signatures of early *Apc^Min/^*^+^ tumours as well as of revival stem cells [156,159]. Accordingly, YAP decorates the nuclei of tubular adenomas from patients afflicted with familial adenomatous polyposis [158]. Yet, the role of YAP in intestinal tumorigenesis remains controversial as both tumour-suppressive [153,155] and oncogenic functions [152,158,160,161] have been ascribed in different contexts. It further remains to be seen whether the foetal-like, YAP/TAZ-dependent, *Lgr5*^−^ regenerative state plays a role in the development of colonic tumours lacking overt Wnt-pathway mutations. In support of this notion, BRAF^V600E^-driven colonic organoids exhibit a foetal-like dedifferentiation program enriched for Hippo-pathway targets, which recapitulates the transcriptional profiles of human BRAF^V600E^-driven CRCs [162], although in vivo validation is currently lacking.

From a therapeutic standpoint, the acquisition of a YAP/TAZ-dependent foetal-like signature reportedly underpins resistance to Wnt-targeted therapy. To recapitulate the CRC mutational landscape, Han and colleagues [163] generated mouse colonic organoids harbouring oncogenic *Ptprk*-*Rspo3* fusions, *Kras^G12D^* or *Braf^V600E^*, and loss-of-function mutations in the tumour-suppressor genes *p53* and *Smad4*. Transient exposure of these organoids to TGFβ, intended to select for *Smad4*-mutant lines, conferred resistance to PORCN inhibition, signifying that the emergent organoids had lost their dependence on Wnt signalling. Similarly to the regenerating epithelium, this WNT-independent growth is associated with YAP/TAZ-dependent transcriptional reprogramming and reversion to a foetal-like state, which crucially retains sensitivity to YAP/TAZ inhibition [163].

A newly discovered upstream regulator of YAP signalling suggests another potential therapeutic vulnerability. PGE2—secreted into the niche by a rare population of PTGS2-expressing pericryptal fibroblasts—drives the expansion of *Sca1*^+^ reserve-like stem cells, with a regenerative/tumorigenic YAP transcriptional signature and concomitant suppression of β-catenin signalling, fuelling adenoma initiation in *Apc^Min/^*^+^ mice as well as an azoxymethane-induced tumour model. Crucially, the tumorigenic capacity of these *Sca1*^+^ reserve-like stem cells depends on the druggable PGE2–PTGER4 axis, which in turn controls the nuclear localization/activity of YAP [159]. Indeed, PGE2-induced YAP signalling is also implicated in colitis-associated regeneration and spontaneous tumorigenesis [164]. These results exemplify the contribution of a proinflammatory mesenchymal niche to tumour initiation. Perhaps more importantly, they suggest a therapeutically actionable target for the pro-oncogenic, YAP-dependent, foetal-like regenerative program.

Overall, multiple cell types can mobilize to regenerate the injured intestinal epithelium by adopting a highly plastic foetal-like state, although the degree to which each population contributes to the repair warrants further study. It also remains unclear whether regenerative cues can mobilize different crypt progenitors and/or mature cell types to dedifferentiate into a foetal-like state, and/or whether pre-existing homeostatic crypt cell types, such as the *Lgr5*^−^*Clu*^+^*Sca1*^+^ revival stem cells [156], expand in an attempt to restore the epithelium independently of Wnt signalling. Furthermore, whether revival stem cells can serve as tumour-initiating cells remains untested at present. For example, it is conceivable that PGE2-dependent *Sca1*^+^ reserve-like tumour-initiating cells [159] derive from transformation of *Lgr5*^−^*Clu*^+^*Sca1*^+^ revival stem cells [156]. Consistent with this notion, the revival stem cell signature correlates with resistance to 5-fluorouracil chemotherapy in patient-derived CRC organoids, and elevated *CLU* expression is associated with poor patient survival and disease recurrence [165]. In addition, the revival stem cell signature is reportedly enriched in L1CAM-positive metastasis-initiating CRC cells [166], a point we return to later.

## 9. Nutritional Cues and ISC Function

Several studies have contended that organismal nutritional status, quality of nutrient intake, and different dietary regimens modulate ISC behaviour and regenerative capacity as well as impact the composition and diversity of the gut microbiota. As the roles of dietary factors and host–microbiota crosstalk in intestinal homeostasis and disease have been extensively reviewed [167,168,169,170,171,172,173], herein we highlight key findings that inform on how nutrients and microbiota contribute to the niche in homeostasis, and how they erode ISC function in tumour progression.

A high-fat diet and obesity promote proliferation of *Lgr5*^+^ and *Lgr5*^−^ ISCs, including progenitor populations, in a PPARδ-dependent manner likely expanding the ISC pool vulnerable to transformation [174]. In this instance, ISCs exhibit β-catenin-dependent upregulation of the Notch ligands JAG1 and JAG2. This cell-autonomous activation of Notch signalling renders ISCs independent of the Paneth cell niche, the source of Notch ligands in non-pathological conditions. Gaining independence from niche signals predisposes to deregulated ISC self-renewal and tumorigenesis, with the corollary that fasting and dietary intervention may influence ISC behaviour, CRC risk, and treatment efficacy. Of note, excess dietary cholesterol also stimulates ISC proliferation and promotes tumour formation in *Apc^Min/^*^+^ mice, but does so directly by impacting phospholipid remodelling and the membrane lipid composition of *Lgr5*^+^ ISCs [175].

A high-fat ketogenic diet promotes *Lgr5*^+^ ISC stemness and regenerative capacity post injury through the activation of Notch signalling [176]. Expression of HMGCS2—the rate-limiting enzyme in the biosynthesis of ketone bodies produced by the breakdown of fat—is highly enriched in *Lgr5*^+^ ISCs relative to differentiated cells. Its product, β-hydroxybutyrate (βOHB), inhibits class 1 histone deacetylase (HDAC) enzymes, leading to the activation of Notch target-gene expression. Accordingly, *Hmgcs2* deletion depleted βOHB levels in *Lgr5*^+^ ISCs, impaired Notch-driven self-renewal potential, and prompted their differentiation towards a secretory cell fate, outcomes which could be prevented by exogenous βOHB or HDAC inhibitors. Importantly, a glucose-supplemented diet phenocopied the effects of *Hmgcs2* deletion: it suppressed *Hmgcs2* expression in *Lgr5*^+^ ISCs, reduced crypt βOHB levels, dampened Notch signalling, and diminished lineage tracing from *Lgr5*^+^ ISCs post irradiation [176]. A ketogenic, low carbohydrate diet, therefore, promotes Notch-pathway activity and sustains a mobilizable pool of *Lgr5*^+^ ISCs that can readily replenish the epithelial lining post injury, whereas a high-sugar diet suppresses ketogenesis and compromises *Lgr5*^+^ ISC regenerative capacity [176].

Recent studies have directly implicated the Western diet—high in fat and low in calcium and vitamin D—in the differential mobilization of distinct ISC pools during homeostasis and tumorigenesis [177]. Wild-type mice fed a Western-style diet (NWD1), formulated with nutrients known to increase the risk of CRC (higher fat; lower vitamin D3, calcium, folate/methionine, and fibre) [178,179], develop both large- and small-intestinal tumours, which recapitulate the incidence and latency of human sporadic CRC [180]. Surprisingly, lineage tracing of emergent tumours in this model showed that they harbour a reduced number of *Lgr5*^+^ cells but an increased proportion of *Bmi1*^+^ cells [181]. During tumour latency, NWD1-fed mice appear normal but nevertheless harbour multiple changes in their histologically normal mucosa, including an enlarged proliferative zone, perturbed cell differentiation patterns, and expanded Wnt signalling throughout the mucosa of their small-intestinal villi and colonic crypts, pointing to a dysfunctional stem cell compartment [180,182]. Indeed, both the repopulating ability and tumour-initiating potential of *Lgr5*^+^ ISCs from NWD1-fed mice were severely impaired [183]. Replenishing higher levels of dietary vitamin D3 restored *Lgr5*^+^ ISC stemness [183]. Deletion of the *Vdr* gene, encoding the vitamin D receptor, recapitulated the effects of the NWD1 diet, with *Lgr5*^+^ cells unable to repopulate the crypt during homeostasis [183] and *Bmi1*^+^ ISCs mobilized in their stead [177]. These studies establish vitamin D as a key ingredient of the nutrient niche, linking *Lgr5*^+^ ISC stemness with physiological gut function. They also suggest further important avenues for research into how and which dietary factors influence the mobilization of different stem cell populations in distinct modes of injury, tumour subtypes, as well as post therapy, which may also inform potential dietary intervention strategies.

The above studies further raise a cautionary note about the differences in nutrient levels between mouse chows and the human diet. For example, mouse chows typically supply higher vitamin D levels than consumed by humans [177,181,183] whereas, in fact, an increased risk of CRC is associated with vitamin D deficiency [184]. Moreover, the reliance of the stem cell function of *Lgr5*^+^ ISCs on vitamin D receptor signalling, coupled with the fact that *Lgr5*^+^ ISCs enter quiescence in its absence, raises the provocative possibility that *Lgr5*^+^ ISCs may not be as readily mobilizable under diets recapitulating low vitamin D intake that are more relevant to the human condition [183]. It is also noteworthy that the levels of fat (60%), typically administered in so-called high-fat diets [174], are considerably higher than those found in the diets of obese humans, and they confer a markedly more pronounced metabolic response relative to a 45% fat diet, which better recapitulates human physiology [185,186]. These considerations have led some scientists to question whether such high-fat diets provide an appropriate model for human diet-induced obesity [185,186]. In addition, different diets have been shown to profoundly alter the gene expression landscapes of *Lgr5*^+^ ISCs, underscoring the importance of considering the composition of the diet in the experimental design and prompting the recommendation for transparent reporting of dietary context in stem cell studies [187].

Aside from the quality of nutrition, fasting and caloric restriction regimens have enjoyed recent attention on account of their purported benefits in extending lifespan and counteracting age-associated attrition of gut and ISC function [188]. Prolonged calorie restriction elicits a reduction in small-intestinal epithelial mass and stimulates Paneth cell-derived paracrine signalling, priming the niche for rapid *Lgr5*^+^ ISC expansion following nutrient repletion [189]. In contrast, acute nutrient deprivation mobilizes *mTert*^+^ ISCs to repopulate the epithelium upon re-feeding [190], suggesting differential mobilization of ISC pools following distinct types of dietary perturbation. From a therapeutic standpoint, fasting protects *Lgr5*^+^, *Bmi1*^+^, and *HopX*^+^ ISC populations from DNA damage [191] and alleviates chemotherapy-induced toxicity in both mice and patients [191]. Mechanistically, fasting induces a PPARδ-dependent fatty acid oxidation program in ISCs and progenitor cells, which channels triglycerides and free fatty acids into acetyl-CoA/energy production, improving regenerative potential and counteracting the age-associated decline in intestinal function [192].

Given that fasting and a high-fat diet differ drastically with respect to calorie intake, it is surprising that a high-fat diet also activates PPARδ signalling and augments the self-renewal capacity of both *Lgr5*^+^ and *Lgr5*^−^ ISCs [174]. Furthermore, ectopic PPARδ signalling increases the tumour-initiating efficiency of intestinal progenitors following loss of *Apc* [174,193]. In this regard, it is worth considering that prolonged consumption of a high-fat diet exposes ISCs to an excess of dietary fatty acids over the longer term, persistently deregulating β-catenin transcriptional activity and expanding both *Lgr5*^+^ and *Lgr5*^−^ ISCs independently of niche constraints [174]. By contrast, acute fasting mobilizes scant reserves of free fatty acids from adipose stores to restore diminishing ISC function as part of a transient adaptive response to nutrient deprivation [174,192]. Notwithstanding these differences, PPARδ is emerging as a key, druggable metabolic node that controls ISC fate and function in response to nutritional cues, such as changes in nutrient availability and quality. Interestingly, PPARδ was recently implicated in mediating the effects of a high-fat diet on colonic tumour initiation and liver metastasis via the activation of its downstream target, the pluripotency factor NANOG [194], underscoring its pleiotropic modes of action in regulating stemness and tumour progression. Whether aberrant activation of fatty acid oxidation per se contributes to tumorigenesis awaits further investigation [192], although recent studies have linked activation of this pathway to anoikis resistance in metastasizing CRC cells [195]. Together, these findings hold promise for the use of fasting regimens and PPARδ agonists in ameliorating intestinal dysfunction and improving the tolerability and efficacy of current chemotherapies [191,192] but, conversely, advocate the evaluation of PPARδ antagonists in the CRC setting—particularly for obese patients. Interrogating which pathways downstream of PPARδ are activated in distinct settings/CRC subtypes, and distinguishing which PPARδ coactivator/corepressor interacting partners are recruited to its homeostatic roles in normal ISC physiology, and which can impact oncogenesis, may yield valuable therapeutic insights.

## 10. Microbiota-Derived Metabolites and ISC Function

The gut microbiota—a complex ecosystem of bacteria, archaea, eukaryotes, and viruses that inhabit the intestinal tract—benefit from host nutrient intake and, in turn, influence host metabolism, physiology, nutrition, and immune function. The gut microbiota ferment indigestible by-products of host digestion, such as dietary fibre, and metabolize ingested nutrients, xenobiotics, and bile acids to produce energy, short-chain fatty acids, lactate, various vitamins, and other nutritious metabolites [168,169,170,171,172,173].

Ingested dietary nutrients can, therefore, drastically alter the microbiota landscape and metabolic output. In turn, microbiota-derived metabolites can directly impact ISC function and gut homeostasis in multiple ways [168,169,170,171,172,173]. For example, dietary fibre is fermented by commensal bacteria in the colon, generating short-chain fatty acids such as butyrate, a pleiotropic metabolite which functions both as an energy source and an HDAC inhibitor. Of note, butyrate also helps regulate immune homeostasis in the gut. It promotes immune tolerance by downregulating the expression of proinflammatory mediators [196,197], desensitizing lamina propria macrophages to commensal microbiota [196,197], and inducing Treg differentiation [198,199].

Functioning as an HDAC inhibitor, butyrate can suppress the proliferation of ISCs in vitro by enhancing the promoter-binding activity of the cytostatic transcription factor FOXO3 [200]. In vivo, however, bacterial-derived butyrate can only inhibit ISC proliferative capacity following disruption of the crypts by injury as it is normally excluded from the niche. Indeed, during homeostasis, the elaborate crypt configuration ensures that differentiated colonocytes metabolize butyrate, forming a metabolic barrier that restricts the levels of butyrate “trickling down” to the crypt base, thereby shielding vulnerable ISCs from its cytostatic effects [200]. The ability of butyrate to inhibit ISC proliferation comes into play, however, following colonic mucosal injury. By inhibiting ISC proliferation, butyrate delays the regenerative response until the wound is sealed, reducing the risk of exposing proliferating *Lgr5*^+^ ISCs to bacterial genotoxins, which could cause DNA damage and elicit their transformation [200].

Notably, butyrate exerts differential effects on proliferation and apoptosis in normal versus cancerous colonocytes, a conundrum known as the “butyrate paradox” [201,202]. Whereas normal, differentiated colonocytes metabolize butyrate as an energy source to sustain proliferation [203], cancerous colonocytes opt for aerobic glycolysis, on account of the Warburg effect, leading to the accumulation of butyrate [204]. In this context, the excess butyrate functions as an HDAC inhibitor, causing hyperacetylation of core histones and leading to the expression of genes whose products inhibit cell proliferation and induce apoptosis [201,204]. Although widely regarded, therefore, as a tumour-suppressive metabolite [196,202], butyrate has also been shown to exert pro-tumorigenic effects further adding to its namesake paradox. Belcheva et al. found that butyrate fuels colonocyte proliferation and promotes polyp formation in an *Apc^Min/^*^+^*Msh2*^−/−^ mouse model, potentially linking butyrate-producing commensal bacteria to the pathogenesis of CRC [205]. Differences in the host genetic background and age, gut microbiota status, chow formulation/dietary fibre source, tumour subsite location within the colon, and the local metabolite milieu (e.g., the presence of interacting metabolites) may all contribute to this apparent paradox [206,207]. Nevertheless, this context-dependence lends hope for the development of antibiotic and dietary interventions that could harness the microbiome to influence disease progression and improve therapeutic outcome.

Secondary bile acids are another important class of metabolites that are produced by the gut microbiota from unabsorbed primary bile acids. They facilitate absorption of dietary fats and fat-soluble vitamins while also serving as pleiotropic signalling molecules through the activation of both their cognate nuclear receptor, FXR [208], and the G protein-coupled bile acid receptor, TGR5 [209]. Elevated faecal levels of secondary bile acids, particularly deoxycholic acid and lithocholic acid, are associated with the consumption of a high-fat/high-protein and low complex carbohydrate diet, and correlate with an increased predisposition to CRC [210,211]. Directly linking high-fat diet-induced accumulation of bile acids to tumour initiation, excess colonic bile acids have been shown to erode the crypt–villus architecture in mice, perturbing locoregional Wnt-signalling gradients and exposing *Lgr5*^+^ ISCs to luminal genotoxins and ensuing transformation [208]. Feeding *Apc^Min/^*^+^ mice a high-fat diet shifted bile acid metabolism toward the increased production of tauro-β-muricholic acid and deoxycholic acid, which antagonize the function of the bile acid receptor FXR, stimulating *Lgr5*^+^ ISC proliferation, causing DNA damage and genomic instability, and facilitating adenocarcinoma progression. Notably, these effects could be curtailed by FXR agonists, which are currently under clinical evaluation [208].

Bile acids also relay nutrient availability by activating the receptor TGR5 in *Lgr5*^+^ ISCs, thus coordinating daily intestinal epithelial turnover post food intake. Release of bile acids into the intestinal lumen is sufficient to stimulate *Lgr5*^+^ ISC self-renewal, TA progenitor proliferation, and specification into the goblet and enteroendocrine cell lineages via TGR5 [209]. Loss of TGR5 function in *Lgr5*^+^ ISCs exacerbated DSS-induced damage and impaired the regenerative capacity of the colonic epithelium, implicating bile acid–TGR5 signalling in regeneration post injury [209]. Mechanistically, the bile acid–TGR5 axis promotes intestinal regeneration via SRC/YAP signalling and engagement of the foetal regenerative program [149,209]. Together, these findings cast bile acids as potent, pleiotropic oncometabolites that can influence both *Lgr5*^+^ and *Lgr5*^−^ populations, expanding the potential tumour-initiating pool.

Microbiota-derived lactate has also been shown to promote *Lgr5*^+^ ISC proliferation, albeit indirectly, by enhancing Wnt/β-catenin signalling in Paneth cells and αSMA^+^ intestinal stromal cells via the GPR81 receptor [212]. Typically, the highly glycolytic Paneth cells in the small intestine supply neighbouring *Lgr5^+^* ISCs with lactate, used to fuel oxidative phosphorylation and sustain their prolific mitochondrial metabolism [115]. Rather than altering metabolic output, however, it seems that commensal microbiota-derived lactate may serve to reinforce the nurturing functions of Paneth and stromal cells, particularly by stimulating the increased secretion of WNT3, to support the regenerative capacity of *Lgr5*^+^ ISCs [212]. Interestingly, oral administration of lactate or probiotics protects mice from radiation- and chemotherapy-induced intestinal damage, which argues in favour of their prophylactic administration to ameliorate gut injury sustained in response to genotoxic therapy [212].

Although not a microbiota-derived metabolite per se, arachidonic acid deserves mention as an essential dietary fatty acid with broad anti-microbial properties that may influence both the microbiota landscape [213] and ISC function [214]. As such, arachidonic acid is thought to selectively favour the growth of commensal bacteria but suppress the proliferation of pathogenic microbiota [213], in addition to enhancing the proliferative and regenerative response of the small-intestinal epithelium to irradiation [214]. Mechanistically, arachidonic acid elevates the expression of *Ascl2*, the master regulator of Wnt signalling [73,214]. Yet, interestingly, arachidonic acid exerts its pro-regenerative effects on the recently described *Msi1*^+^ radioresistant ISC pool [45,214], rather than *Lgr5*^+^ ISCs, which are in fact depleted by arachidonic acid treatment [214].

Overall, the above findings establish dietary nutrients and microbiota-derived metabolites as important influencers of stemness with the capacity to mobilize both *Lgr5*^+^ and *Lgr5*^−^ populations, confer independence from the niche, and expand the cell pool vulnerable to transformation. An improved understanding of how ingested nutrients and microbiota-derived metabolites influence the function of the various ISC subpopulations may inform the development of effective dietary or pharmacologic intervention strategies to mitigate CRC risk. Additionally, new insights may lead to the development of probiotics and dietary supplements as potential adjunct regimens for improving the tolerability and efficacy of CRC therapies.

## 11. Microbiota Contributions to the Niche

The evidence discussed above converges on the pivotal roles of the intestinal microbiota and their metabolites in influencing ISC function during homeostasis, post-injury regeneration, and CRC progression. The intestinal microbiota can impact each of these processes in multiple different ways. These include curtailing or promoting inflammation, impairing or reinforcing gut-barrier integrity, perturbing invasive bacterial species from colonizing the gut, influencing the host immune response, generating genotoxins that damage the host DNA, modulating the balance between ISC self-renewal and differentiation, and altering the metabolic milieu (reviewed in [167,168,169,170,171,173]). Moreover, dysbiosis—an imbalance of the commensal and pathogenic microbial communities in the gut—has been increasingly recognized as a prelude to colonic inflammation [215] and a catalyst of inflammation-associated CRC initiation and progression [167,216,217,218,219]. 

The microbiota composition and abundance differ significantly along contiguous intestinal segments, with the large intestine/colon typically displaying a higher bacterial burden and species diversity compared with the small intestine [220]. In addition, distinct taxa colonize diverse regions along the tissue–lumen axis as influenced by locoregional nutrient availability, oxygen gradients, pH conditions, and metabolic specialization [220,221]. As a result, microbiota-derived metabolites also vary regionally creating multiple local metabolite milieux that can profoundly impact gut physiology and tumorigenesis [220,221]. Although the majority of gut microbiota reside either in the lumen or within the mucus layer above the villi, a select contingent of aerobic, non-fermentative genera (*Acinetobacter*, *Stenotrophomonas*, and *Delftia*) inhabit murine ceacal and proximal colonic crypts [222]. These so-called “crypt-specific core microbiota” augment the homeostatic function of closely apposed *Lgr5*^+^ ISCs [223]. Recently, an analogous community of crypt-specific core microbiota has been reported to colonize human colonic crypts and to become dysbiotic in CRC [224].

The presence of microbial components can directly impact the proliferation and differentiation of ISCs and affect their role in preserving the integrity of the epithelial barrier. *Lgr5*^+^ ISCs are equipped with both cell-surface (TLR4) and intracellular (NOD2) pattern recognition receptors that enable the detection of shed bacterial components. In the small intestine, engagement of TLR4 by its bacterial cell wall-derived ligand, lipopolysaccharide, inhibited the proliferation of *Lgr5*^+^ ISCs and induced PUMA-dependent apoptosis, suggesting that invading pathogens may directly target *Lgr5*^+^ ISCs and perturb their ability to regenerate the epithelium post infection [225]. Having identified that colonic crypts harbour the so-called crypt-specific core microbiota [222], Naito and colleagues assessed how their presence impacts colonic ISC function in homeostasis [223]. Monocolonization of germ-free mice with individual members of the crypt-specific core microbiota [222] activated TLR4 signalling, leading to a shift toward goblet cell differentiation and eliciting proinflammatory necroptosis of ISCs and TA cells [223], in contrast to the small intestine where immunosuppressive apoptosis was the predominant mode of cell death [225]. TLR4-induced necroptosis of ISCs may therefore help maintain crypt homeostasis by regulating the balance between cell proliferation and differentiation in the colonic epithelium, but it may also serve to initiate an antimicrobial inflammatory response to translocated microbiota [223]. Notably, excessive TLR4 signalling delays mucosal healing and precipitates a pronounced inflammatory storm as seen when the immature immune system encounters bacterial translocation in necrotizing enterocolitis, a leading cause of death in premature infants [225].

*Lgr5*^+^ ISCs also express the intracellular innate immune NOD2 receptor, which can detect the muramyl dipeptide (MDP) motif of peptidoglycan, another component of the bacterial cell wall [226]. Following irradiation, binding of MDP to NOD2 recruits the autophagy effector ATG16L1, which instigates the removal of damaged mitochondria and harmful reactive oxygen species by mitophagy. Consequently, MDP-induced NOD2 signalling enhances ISC survival and preserves regenerative capacity without evoking a detrimental inflammatory response [226,227]. Interestingly, another study showed that *Bmi1*^+^ +4/reserve ISCs were depleted in irradiated *Nod2*^−/−^ mice, suggesting that NOD2 not only protects *Lgr5*^+^ ISCs from damage but also safeguards *Bmi1*^+^ +4/reserve cells [228]. Failure to engage MDP–NOD2 signalling could therefore severely compromise the regenerative ability of the intestinal epithelium and perpetuate inflammatory damage, as exemplified in Crohn’s disease, frequently associated with *NOD2* mutations or polymorphisms [227]. Thus, microbe-associated molecular patterns impact the function of ISCs in profoundly different ways. How ISCs distinguish between commensal and pathogenic bacteria, however, remains unclear. 

Commensal microbes can also help restore ISC function following inflammatory assault. For example, the probiotic strain *Lactobacillus reuteri D8* augments the proliferation of *Lgr5*^+^ ISCs and Paneth cells, ameliorating the damage caused by TNFα in small-intestinal organoids and by DSS in mice [229]. Sensing of the *L. reuteri* metabolite indole-3-aldehyde, by the AhR receptor on co-cultured human lamina propria lymphocytes, stimulated secretion of IL22, driving pro-proliferative STAT3 signalling in *Lgr5*^+^ ISCs and Paneth cells [229]. *L. reuteri* also stimulated the proliferation of *Lgr5*^+^ ISCs by elevating RSPO expression and activating Wnt/β-catenin signalling [230]. In the setting of TNFα-induced inflammation, the presence of *L.* reuteri ensured that the number of *Lgr5*^+^ ISCs was maintained, concomitant with the induction of Paneth cell differentiation, leading to the release of antimicrobials that restricted colonization by the attaching and effacing pathogen *Citrobacter rodentium* [230]. These findings exemplify how commensal bacteria and their metabolites can impact ISC homeostasis and reinforce the epithelial barrier against inflammatory insult and pathogen infection. Future studies will no doubt shed light on the key gut microbiota–host interactions and immune cell circuits, involved in regulating the maintenance and activity of different ISC populations in homeostasis and disease, and address whether these can be harnessed for therapeutic benefit.

Few studies to date have examined how pathogenic bacterial invasion impacts ISCs and their niche. A recent study showed that the enteric pathogen *Clostridioides difficile* deploys the exotoxin TcdB to disrupt epithelial polarity and crypt architecture by targeting β-catenin/E-cadherin complexes and the scaffold protein Ezrin. Indeed, *C. difficile* causes damage deep into the epithelium, exposing the *Lgr5*^+^ ISCs that are ordinarily protected at the crypt base. Pathogenic infection of *Lgr5*^+^ ISCs attenuates Wnt signalling and impairs their ability to regenerate the injured epithelium, exacerbating tissue damage, delaying recovery, and predisposing to re-infection [231]. In a *Salmonella enterica* colitis model, the pleiotropic bacterial effector AvrA dampens proapoptotic innate immune responses and suppresses inflammation [232] while paradoxically activating Wnt/β-catenin signalling to increase the number of ISCs and proliferating cells [233]—all part of an elaborate strategy to evade host immune defences and maintain the ISC niche so as to ensure long-term survival of the pathogen within the host [233]. Such chronic *Salmonella* infections can increase the risk of CRC. Relative to AvrA^−^ counterparts, AvrA^+^ *Salmonella typhimirium* significantly increased tumour incidence in an inflammation-associated azoxymethane/DSS colon cancer model, intriguingly shifting the tumour topography from the distal (left) to the proximal (right) colon [234]. Emergent tumours in this model display activation of β-catenin signalling and elevated expression of the BMI1 stem cell marker, consistent with the notion that the AvrA effector subverts host ISC-signalling pathways [234].

As mentioned above, CRC progression is often accompanied by pronounced changes in the abundance, composition, and diversity of microbiota both in the tumour and the adjacent mucosa, termed dysbiosis [167,216,217,218]. In fact, changes in the microbiota and their associated metabolome are detectable from the very early stages of CRC, implicating such dysbiotic changes in disease aetiology [235]. Interestingly, distinct microbiota profiles associate preferentially with each CRC subtype [236,237], and the composition of the microbiota differs significantly between right- and left-sided colon tumours [219,224,238], likely reflecting the profound differences in the underlying disease pathology. For example, one study found that *Fusobacterium periodonticum* and *Bacteroides fragilis* were more prevalent in right-sided tumours, whereas *Parvimonas micra* dominated left-sided lesions [224]. Indeed, right-sided CRCs emerge proximal (right) to the splenic flexure in a very different milieu, compared with left-sided CRCs (distal), and are thought to progress through an alternative serrated neoplasia pathway, molecularly underpinned by mutations in *BRAF* or *KRAS*, microsatellite instability, and a CpG island methylator phenotype [239,240]. Of note, patients with right-sided/proximal colon cancer typically harbour invasive multi-microbial communities encased in a complex protective ECM—termed biofilms—both in their tumour mucosa and the tumour-distant normal mucosa [238], implicating such biofilms and the inflammation they incite in the pathogenesis of right-sided disease [167,238]. The presence of biofilms on the normal mucosa of sporadic CRC patients is believed to indicate a tissue primed for tumorigenesis [238]. Similar colonic biofilms, enriched for *Escherichia coli* and *B. fragilis*, have also been detected in patients with familial adenomatous polyposis [241].

The depletion of gut microbiota through antibiotic treatment markedly attenuates tumour burden in multiple models, and germ-free animals are resistant to intestinal tumorigenesis [205,242,243,244,245,246], reinforcing the notion that the development of CRC is influenced by microbiota–host crosstalk. Several bacterial species have been implicated in promoting tumorigenesis in humans such as *Fusobacterium nucleatum* [247,248,249], *E. coli* that produce the genotoxin colibactin [241,245], and enterotoxigenic *B. fragilis* [241,250]. For example, the abundance of *F. nucleatum* increases along the adenoma-carcinoma sequence [247,248], and its enrichment correlates with poor patient survival [249]. Mechanistically, *F. nucleatum* deploys the FadA adhesin, a virulence factor which binds E-cadherin thereby facilitating host-cell bacterial invasion, activation of Wnt/β-catenin signalling, and the induction of proinflammatory genes [251]. Remarkably, *F. nucleatum*, and its associated *Bacteroides*, *Selenomonas*, and *Prevotella* species, have been detected in distal metastatic lesions as well as in their respective primary tumours, with the corollary that *F. nucleatum* and its associated microbiota may form a “mobile niche”, which disseminates aboard circulating tumour cells to the distant site while abetting metastatic competence [246]. Colorectal tumours with a high *F. nucleatum* load are also more likely to recur post chemotherapy, with the bacteria able to exploit TLR4 and MYD88 innate immune signalling to induce autophagic survival and resistance to chemotherapy [252]. Encouragingly, treatment of xenograft-bearing mice with the antibiotic metronidazole reduced the *F. nucleatum* burden and compromised cell proliferation and tumour growth, advocating for the development of antimicrobial therapeutic interventions for *F. nucleatum*-associated CRC [246].

Several recent studies have further underscored the links between dysbiosis and CRC. Mice lacking the cytoplasmic, innate immune, double-stranded DNA sensor AIM2 show aberrant Wnt signalling and an expansion of *Prox1*^+^ ISCs, predisposing to colonic tumour development following treatment with azoxymethane and DSS [253]. *Aim2^−/−^* mice harbour dysbiotic microbiota comprising multiple CRC-associated species and, strikingly, their tumour burden was considerably decreased upon exposure to gut microbiota from co-housed wild-type mice. Conversely, the colonic tumour burden of the co-housed wild-type mice was increased, compared with individually housed wild-type counterparts, implicating dysregulation of transmissible microbiota as an underlying cause [253]. These findings implicate AIM2 in the protection against inflammation-associated tumorigenesis through its ability to modulate the gut microbiota and suggest that microbiota engraftment may help limit tumour development in CRC patients with loss-of-function mutations in *AIM2* [253].

A further landmark study recently established a driver role for colibactin-producing bacteria in CRC tumorigenesis. Whole-genome sequencing of organoids, repeatedly exposed to colibactin-producing *pks*^+^ *E. coli*, revealed that emergent cells harboured two distinctive mutational signatures characteristic of human CRC, strongly implicating *pks*^+^ *E. coli* and colibactin-induced genotoxic damage in CRC development and progression [254]. In another intriguing recent study, Kadosh et al. reported that the regional gut microbiota and, in particular, high levels of the bacterial-derived metabolite gallic acid altered the phenotype of mutant p53 from a tumour suppressor to an oncogene in two models of Wnt-driven colon cancer [255]. The functionality of mutant p53 varied depending on the location of the cells along the length of the intestine, indicating that p53 oncogenic and tumour-suppressive functions may be more pliable than generally thought. In the proximal gut of *CKIa^Δgut^* and *Apc^Min/^*^+^ mice, mutant p53 (p53^R172H^ or p53^R270H^) surprisingly suppressed the development of dysplasia and tumorigenesis, respectively, by counteracting Wnt-driven hyperproliferation. In the distal gut, however, mutant p53 switched to an oncogenic mode of action whereby it hyper-activated Wnt signalling and enhanced tumorigenesis [255]. Eradication of the gut microbiota, by treatment with antibiotics, reduced Wnt activity, decreased cell proliferation, and prevented the onset of dysplasia in the ileum and colon of *CKIa^Δgut^p53^R172H^* mice, suggesting that the regional microbiota likely counter the tumour-suppressive activity of mutant p53 in the distal gut thereby promoting tumorigenesis. Remarkably, supplementation of gallic acid—a polyphenol metabolite produced by *Lactobacillus plantarum* and *Bacillus subtilis* in the distal, but not the proximal gut—was sufficient to neutralise the tumour-suppressive ability of mutant p53 and unleash its oncogenic activity. Accordingly, gallic acid treatment led to high-grade dysplasia in the proximal gut of *CKIa^Δgut^p53^R172H^* and *Apc^Min/^*^+^*p53^R172H^* mice and restored tumour formation to the distal gut of antibiotic-treated *CKIa^Δgut^p53^R172H^* mice [255]. While key mechanistic questions remain, these studies raise the possibility of targeting the bioactivity and/or bioavailability of gallic acid for Wnt-driven CRCs harbouring mutant p53.

Another important advance is the development of the first microbiota-dependent spontaneous invasive tumour model. Mice engineered to express intestinal epithelial cell-specific ZEB2—a transcription factor that orchestrates the epithelial-mesenchymal transition (EMT)—exhibited increased barrier permeability, dysbiosis, and myeloid cell-driven inflammation, leading to the development of invasive colonic tumours in a microbiota-dependent manner [256]. Underscoring the importance of a dysbiotic milieu, the small-intestinal epithelium did not show evidence of dysplasia, and antibiotic-mediated depletion of the microbiota or germ-free rederivation abrogated tumour formation in this model [256]. Although the authors stopped short of characterizing the dysbiotic species underlying tumour development in this model, the identification of the driver species and/or their key oncometabolites will provide valuable mechanistic insights into dysbiosis-driven CRC.

To conclude, diverse and regionalised microbiota, their secreted metabolites, and their virulence factors and effector proteins converge with host-derived immune cells, ingested nutrients, cytokines, and growth factors to influence niche-signalling and ISC function in homeostasis, post-injury regeneration, and CRC. Altogether, the abovementioned compelling findings reinforce the notion that gut microbiota can profoundly influence tumorigenesis and are an integral part of the niche/tumour microenvironment (TME), influencing the growth of emergent tumours in concert with the genetic make-up of the tumour cells. They also open up the realm of manipulating the microbial and metabolite milieu for therapeutic gain, albeit with the humbling realisation that we still have much to learn about harnessing the crosstalk between the microbial ecosystem and the wider niche/TME. 

## 12. ISC Dynamics in the Niche: “Winner Takes All”

Mouse ISCs divide symmetrically generating equipotent progeny that stochastically compete for limited niche space via “neutral-drift” kinetics, whereby each descendant has an equal probability of achieving clonal dominance over its neighbours [257,258]. Intravital imaging, however, elegantly showed that so-called “central ISCs” are three times more likely to colonize a crypt than “border ISCs”, suggesting that close proximity to the niche at the crypt base is pivotal to the ability of an ISC to colonize an entire crypt (fixation) [259]. Border ISCs are more easily displaced from the niche and are, hence, lost from the ISC pool as they commit to differentiation, with the corollary that if differentiated cells descend back into the niche, they can regain stemness. Interestingly, Paneth cell-secreted WNT3 does not diffuse freely within the niche; instead, FZD-bound WNT3 levels are progressively diluted through receptor-mediated endocytosis and *Lgr5*^+^ ISC division [260]. Thus, the availability of WNT3 becomes limiting further from the crypt base, dictating the size of the niche and differentially imparting distinct stemness capabilities to centre and border ISCs [259]. Consequently, only 5–7 [261,262] of the 14–16 *Lgr5*^+^ cells per crypt [258] manifest long-term self-renewal potential, consistent with the reported heterogeneity within the *Lgr5*^+^ population [30,43,44].

In the mouse SI, time-to-monoclonality (the emergence of monoclonal crypts descended from a single ISC) is 1–6 months [257,258]. While human colonic crypts contain 5–7 functional ISCs—similarly to mouse small-intestinal counterparts—the average time-to-monoclonality is in the order of six years [263,264]. In human colonic crypts, the predominant mode of ISC division is asymmetric, accounting for the sluggish rate of fixation [265]. In addition, human ISCs may also proliferate at an intrinsically slower rate, as observed in xenografted organoids [266], and are likely to be exposed to different environmental cues and dietary nutrients compared with mouse counterparts [177,267]. Nevertheless, while the number of functional ISCs per crypt is comparable between mice and humans, the rate of stochastic replacement of ISCs by their neighbours is significantly lower in human colonic crypts, rendering fixation inefficient over the longer term [264,265].

These population dynamics ensure that expansion of a particular ISC-derived clone is balanced by the extinction of its neighbours, thus maintaining the size of the homeostatic ISC pool constant over time. Mutant cells may be stochastically extinguished from the ISC pool before they can accumulate additional hits, limiting aberrant expansion of mutant lineages [262]. Indeed, caloric restriction—a dietary intervention touted for its anti-cancer benefits and known to reduce intestinal polyp formation in *APC^Min/^*^+^ mice [268]—increases the number of wild-type ISCs competing for niche occupancy, thereby decreasing the likelihood that mutant ISCs will be aberrantly retained in the stem cell pool [269].

During colorectal adenoma-carcinoma progression, however, crypt dynamics are subverted towards a “biased-drift” pattern. Mutations in *Apc* or *Kras*, and/or limited availability of WNT ligands in the niche, confer a competitive advantage on mutant *Lgr5*^+^ ISCs, thereby increasing their likelihood of displacing wild-type counterparts to achieve clonal dominance and fixation [63,262,270,271]. Interestingly, *Kras*-mutant crypts exhibit an increased tendency to undergo fission, whereby the crypt epithelium bifurcates and redistributes the Paneth cells/niche into the two daughter crypts, ensuring the expansion of the *Kras*-mutant epithelium beyond the confines of a single crypt [270]. Increased crypt fission also underlies “field cancerization” (the emergence, in the non-dysplastic mucosa, of patches of crypts harbouring pro-oncogenic mutations) as well as the eventual formation of adenomas initiated from mouse APC-deficient *Lgr5*^+^ ISCs [272]. Indeed, such events are commonly observed in familial adenomatous polyposis [273]. Moreover, although *p53* mutations bear no impact on crypt dynamics during homeostasis, they selectively confer a survival advantage over non-mutant ISCs in the hypoxic inflamed colitis mucosa, which also predisposes to tumorigenesis [262]. These findings demonstrate how pro-oncogenic mutations and changes in the niche/microenvironment cooperate to alter crypt dynamics, accelerate clonal fixation, and increase the frequency of crypt-fission events, promoting adenoma progression.

## 13. Cells-of-Origin

Well- and moderately differentiated colorectal tumours retain some aspects of the glandular architecture and cellular hierarchy of the normal intestinal mucosa [274]. Indeed, analogous to normal crypts, current dogma posits that only a subset of intestinal tumour cells—termed cancer stem cells (CSCs)—are endowed with tumour-initiating potential, i.e., the capacity to self-renew and generate the differentiated non-CSCs that constitute the tumour bulk. CSCs are also thought to underpin metastatic competence, drug resistance, disease recurrence and, ultimately, poor therapeutic outcome. Multiple cell-surface proteins, e.g., CD133 (also known as PROM1), CD166, or CD44, have been proposed to identify distinct subsets of human colorectal CSCs, likely linking the TME with intracellular cancer-driver pathways. However, these are beyond the scope of this article and the reader is referred to recent excellent reviews [275,276,277,278,279,280]. Notwithstanding the CSC hypothesis, and in line with the pervasive plasticity of the intestinal epithelium, accumulating evidence supports both “bottom-up” [273] and “top-down” [281] histogenesis of colorectal tumours whereby the cells-of-origin comprise either ISCs at the crypt base or differentiated cells at the crypt apex, respectively (Figure 2c).

*Lgr5*^+^ ISCs have been amply demonstrated to serve as tumour-initiating cells [59,282]. Indeed, targeted deletion of *Apc* in *Lgr5*^+^ ISCs drives aberrant Wnt signalling and hyperproliferation, leading to rapid adenoma formation in mice [59]. Moreover, overexpression of *Rspo3* in *Lgr5*^+^ cells drives hyperplastic bottom-up lesions, containing mislocalized Paneth cells and expanded *Lgr5*^+^ and *Lgr4*^+^ populations, in keeping with the fact that RSPO3 is a secreted protein that nurtures both *Lgr5*^+^ ISCs and their supportive epithelial niche. Besides *Lgr5*^+^ cells, however, lineage tracing implicates *Lgr5*^−^ populations as putative cells-of-origin of the resulting hyperplastic adenomas and adenocarcinomas in this model [282]. Similarly, constitutive activation of Wnt signalling in cells expressing *Bmi1* [12], *Prom1* [283], or *Lrig1* [15,284] drives bottom-up intestinal neoplasia in mice. Collectively, these findings suggest the existence of multiple possible cells-of-origin within the crypt.

Other crypt cell types can also assume the mantle of tumour-initiating cell, contributing to bottom-up tumorigenesis. Hence, loss of *Apc* in *Krt15*^+^ cells—a heterogeneous population, encompassing *Lgr5*^+^ and *Lgr*5^−^ cells, spanning the crypt base as well as the TA zone—leads to adenomas that occasionally progress to invasive adenocarcinomas [44]. Such lesions are not typically observed upon sole deletion of *Apc* in other putative tumour-initiating cell populations, including *Lgr5*^+^ ISCs [15,18,59,284]. It remains to be determined whether the coexistence of adenomas and adenocarcinomas—frequently a feature of human polyposis syndromes—reflects tumour initiation from distinct differentially localized subsets of *Krt15*^+^ cells [44]. Remarkably, while the majority of *Lgr5*^+^ subsets are exquisitely sensitive to DNA damage, *Krt15*^+^*Lgr5*^+^ cells are radioresistant and may thus survive to spawn tumours post injury.

Conversely, top-down lesions likely derive from cells located in the TA zone or the villus, induced to undergo dedifferentiation. Notably, sole deletion of *Apc* in TA cells yields only microscopic lesions, which rarely progress to adenoma [59]. Additional TGFβ dysfunction is not sufficient to drive dedifferentiation in this compartment or the formation of top-down lesions [285]. However, following exposure to inflammation and/or upon accumulating cooperating mutations, differentiated villus cells can re-express *Lgr5* and ISC markers, and initiate tumours [286,287]. Thus, constitutive activation of β-catenin and NFκB signalling [286] or dual *Apc*/*Kras* mutations [287] can drive tumour formation both from crypt ISCs and villus epithelial cells in the small intestine [286] and colon [287], respectively. Deletion of *Tgfbr1* further augments the dedifferentiation potential of *VilCre^ER^Apc^fl/fl^Kras^G12D/^*^+^ villus epithelial cells, exacerbating top-down tumorigenesis [285]. This suggests that, during early tumour progression, the elevated stromal-derived TGFβ levels that prevail further up the crypt–villus axis restrain dedifferentiation, whereas cells in lower regions or the crypt base can escape to form tumours. Consequently, mutations enabling differentiated cells to evade TGFβ-mediated tumour suppression will extend the pool of tumour-initiating cells. Importantly, the aggressive top-down tumours that emerge, following *Tgfbr1* deletion, exhibit deregulated MAPK signalling and are therefore sensitive to MEK1/2 inhibition, providing an opportunity for early therapeutic intervention [285].

Interestingly, concurrent activation of Wnt signalling and loss of *Smad4* is sufficient to drive dedifferentiation and adenoma formation from enterocytes [288], in contrast to the combined deletion of *Apc*/*Tgfbr1* [285], discussed above. This suggests that SMAD4 may function downstream of BMPs, rather than TGFβ, in restraining epithelial dedifferentiation [288]. Notably, deletion of *Smad4* in the untransformed intestinal epithelium elicits a pronounced TNF-mediated inflammatory response that is sufficient to drive colitis-associated carcinomas. These tumours strongly resemble human *SMAD4*-deficient ulcerative colitis-associated CRCs, thus implicating TGFβ/BMP signalling in the suppression of the innate immune mechanisms that become derailed in colitis [289].

Additional examples whereby dedifferentiation bestows tumorigenic potential have been reported. Long-lived differentiated *Dclk1*^+^ tuft cells, which remain quiescent following *Apc* loss, are readily transformed by inflammation, forming poorly differentiated colonic adenocarcinomas [32]. APC truncation or post-irradiation depletion of *Lgr5*^+^ ISCs induces radioresistant *Krt19*^+^*Lgr5*^−^ upper-crypt progenitors to dedifferentiate, via an *Lgr5*^+^ state, spawning tumours both in the small intestine and colon [18]. *Bhlha15*^+^ secretory cell precursors are another candidate cell-of-origin located just above the ISC zone. In the small intestine, these cells can dedifferentiate to form tumours with serrated features upon sustained activation of Notch signalling, combined with *Apc* loss. In the colon, the counterpart *Bhlha15*^+^ cell population is mobilized upon DSS treatment via the activation of SRC and YAP [290]. Thus, *Bhlha15*^+^ secretory cell precursors respond differently to tumorigenic insult in distinct niches, although the clinical relevance of these findings remains unclear [290].

As mentioned earlier, aberrant expression of the BMP inhibitor *Grem1* in the intestinal epithelium disrupts homeostatic morphogen gradients, prompting the proliferative expansion of *Lgr5*^−^ progenitor cells that spur the formation of ectopic crypt foci perpendicular to the villus axis. Cells, within these structures, accumulate multiple somatic mutations, with concomitant suppression of cytostatic and differentiation programs, eventually progressing to polyps that recapitulate features of hereditary mixed polyposis syndrome and traditional serrated adenomas [94]. These findings further confirm that *Lgr5*^−^ cells, outwith the ISC niche, can undergo malignant transformation.

Together, the above findings reinforce the links between deregulated niche signalling, prolonged inflammation, and CRC risk/progression [291] (Figure 2), and reveal how field cancerization can significantly expand the array of potential tumour-initiating cells [264]. Crucially, they underscore that the initiation of tumours from differentiated cells requires cooperating mutations or exacerbating stimuli, such as an inflammatory drive, alongside Wnt deregulation.

## 14. All Roads Lead through LGR5

Notwithstanding the existence of multiple putative cells-of-origin within the crypt base or more differentiated luminal regions, compelling evidence supports the contention that LGR5 marks a subset of mouse and human intestinal CSCs endowed with tumorigenic potential and multi-lineage differentiation capacity [59,292,293,294,295,296,297,298]. Perhaps unsurprisingly, considering the pervasive plasticity of the intestinal epithelium, ablation of *Lgr5^DTR^* CSCs failed to achieve regression of non-metastatic *Apc^Min/^*^+^*Kras^LSL-G12D/^*^+^*Vil1^Cre^p53*^−/−^*Lgr5^DTR/eGFP^* subcutaneous organoid allografts. Instead, tumours remained in a state of stasis while *Lgr5*^−^ populations mobilized to sustain growth, albeit less efficiently than *Lgr5*^+^ counterparts [297]. Notably, tumour growth resumed unabated following treatment withdrawal, underpinned by dynamic conversion of *Lgr5*^−^ non-CSCs into *Lgr5*^+^ cells. Intriguingly, comparable growth dynamics were observed in cultured organoids, suggesting that the repopulation of *Lgr5*^+^ cells may partly rely on intrinsic *Lgr5*^−^ cell properties and proceed independently of tumour-activated stroma [297]. The mechanisms whereby non-CSCs, or distinct subsets thereof, sense the depletion of *Lgr5*^+^ CSCs within a tumour, and the intrinsic and extrinsic cues that trigger their mobilization remain an important avenue for investigation to better understand therapy resistance and tumour recurrence.

Similarly, xenografted patient-derived organoids contain differentiated *KRT20*^+^ cells that can re-express *LGR5* and fuel tumour regrowth [299]. In this model, short-term ablation of *LGR5*^+^ cells, in combination with anti-EGFR therapy, elicited a more pronounced inhibition of tumour growth than either treatment alone [299]. Consistent with this, residual drug-resistant *LGR5*^−^ cells that can reconstitute tumour growth, following *LGR5*^+^ cell depletion, express the EGF-family member EREG [300]. Interestingly, oxaliplatin did not synergize with anti-EGFR, owing to the failure of chemotherapy to induce *LGR5* expression in *LGR5*^−^ cells [299]. Since *LGR5*^+^ and *KRT20*^+^ cells appear to reside within distinct tumour niches [298], it is plausible that depletion of the *LGR5*^+^ population exposes differentiated *KRT20*^+^ cells to aberrant instructive signals that incite their dedifferentiation and acquisition of CSC traits, analogous to the reversion of multiple intestinal cell types to an *Lgr*5^+^ state during injury-induced regeneration [9,20,26].

Until recently, little was known about the identity of metastasis-initiating cells in CRC and their relationship to primary tumour CSCs. Selective ablation of *Lgr5^DTR^* CSCs in orthotopically implanted *Apc^Min/^*^+^*Kras^LSL-G12D/^*^+^*Vil1^Cre^p53*^−/−^*Smad4*^−/−^*Lgr5^DTR/eGFP^* organoids demonstrated an indispensable role for *Lgr5*^+^ CSCs in the formation and maintenance of metastatic outgrowths, even though their ablation proved inefficacious in the primary tumour setting [297]. Most notably, treatment cessation was not accompanied by regrowth of liver metastases, highlighting the potential therapeutic benefits of targeting *Lgr5*^+^ CSCs in the metastatic setting [297]—the ultimate cause of patient demise. In addition, these findings suggest that distinct tumour cell subsets may harbour differential abilities to drive primary tumour growth and initiate metastases, and underscore the importance of a permissive microenvironment as a prelude for colonization at the distant site [297].

Unexpectedly, ablation of *Lgr5^DTR^* CSCs did not impair primary tumour invasiveness per se, yet still reduced liver metastatic burden, raising the possibility of LGR5-independent mechanisms of productive invasion [297]. Indeed, using intravital multiphoton microscopy to observe spontaneous metastatic progression from orthotopically implanted, genome-edited CRC organoids [301], van Rheenen and colleagues made the striking observation that the majority of circulating tumour cells lacks *Lgr5*. In vitro, *Lgr5*^−^ cells were intrinsically competent to form organoids and spawn functional *Lgr5*^+^ progeny, independently of niche signals, although the emergence of *Lgr5*^+^ cells was increased in the presence of HGF and FGF [302]. Importantly, targeted ablation of *Lgr5^DTR/eGFP^* cells prevented the progression of micrometastases, similar to the findings of de Sousa e Melo et al. [297], with colonization and outgrowth of seeded *Lgr5*^−^ cells dependent on the de novo expression of *Lgr5* [302]. While *Lgr5*^+^ CSCs were detected in the migratory population, they were not typically recovered from the circulation, raising the intriguing possibility that, upon escaping the confines of the primary tumour niche, *Lgr5*^+^ cells enter an *Lgr5*^−^ non-CSC state that likely confers the ability to navigate and survive the perils of the metastatic cascade. Following seeding of *Lgr5*^−^ cells at the distant site, their reversion to an *Lgr5*^+^ state allows the outgrowth and progression of micrometastases. Although organoid cultures suggest that *Lgr5*^−^ non-CSCs can spontaneously revert to an *Lgr5*^+^ state in a niche-independent manner, TME signals can nevertheless influence this transition in vivo. Deciphering the TME signals that instruct the plasticity transitions between *Lgr5*^+^ and *Lgr5*^−^ states, and the underlying molecular mechanisms, may yield important insights into critical determinants of disease progression and therapy resistance, and inform new strategies to target metastatic plasticity.

A further important advance is the identification of the cell-adhesion molecule L1CAM as a cell-surface marker of metastasis-initiating CRC cells [166]. As such, L1CAM is enriched in the invasive front of primary tumours, matched metastases, small cell-clusters invading lymphovascular vessels, and post-therapy surgical resection samples, implicating L1CAM in both metastasis and chemoresistance [166]. Although L1CAM^hi^ cells partially overlap with *LGR5*^+^ CSCs in human CRC organoids, the significance of the various subpopulations, expressing one or both markers, remains unclear.

While L1CAM knockdown does not impact adenoma initiation per se, it significantly inhibits the growth, chemoresistance, and metastatic ability of orthotopically implanted organoids derived from either left-sided *APC*-mutant or right-sided *BRAF*-mutant tumours, underscoring that distinct primary tumour types may nevertheless deploy similar tactics to navigate the metastatic cascade. At the distant site, L1CAM is thought to mediate the heterophilic adhesion of metastasis-initiating cells to the laminin-rich ECM, facilitating colonization and metastatic outgrowth [166].

Interestingly, L1CAM^hi^ metastasis-initiating CRC cells express a gene signature associated with revival stem cells [156] as well as EMT, prompting the authors to draw parallels between the plasticity programs underpinning the regenerative wound-healing response and the disruption of epithelial cell-cell contacts, leading to cell detachment from the primary tumour [166]. Of note, L1CAM has been shown to mediate pericyte-like spreading of disseminated tumour cells on host tissue capillaries by activating YAP [303], a key common denominator of the revival stem cell signature [156] and the regenerative response to injury [149]. Accordingly, while L1CAM is not detected in the homeostatic intestinal epithelium, its expression is markedly induced during processes that disrupt epithelial intercellular contacts, including colitis-associated regeneration and organoid growth [166]. Mechanistically, the disruption of epithelial cell-cell contacts results in the displacement of E-cadherin from the cell membrane, which in turn alleviates REST-mediated repression of the *L1CAM* promoter [166].

Akin to the plasticity of *Lgr5*^−^ disseminated cancer cells that can replenish the damaged *Lgr5*^+^ CSC pool [9,286,297,299] and seed *Lgr5*^+^ liver metastases [302], a subset of L1CAM^lo^ cells can re-express *L1CAM* and engage the regenerative gene expression program during chemotherapy, organoid formation, and DSS-induced regeneration [166]. Organoids and tumours, surviving post chemotherapy, are highly enriched for L1CAM expression, and the combination of irinotecan and L1CAM knockdown is more potently cytotoxic than either treatment alone [166].

While the identification of L1CAM as a metastasis-initiating cell marker in CRC represents an exciting advance, this work raises further questions: Which L1CAM^lo^ cell subsets are poised to switch on *L1CAM* expression? Which TME cues prompt the conversion of the L1CAM^lo^ subsets to L1CAM^hi^ cells, and how can they be targeted to deplete the L1CAM^hi^ cell pool? Is YAP involved in mediating this transition? Does REST de-repression affect entire modules of genes that underpin metastatic competence? Which of the many potential L1CAM binding partners contribute to metastatic competence? Can L1CAM be used prospectively to predict treatment response and metastatic propensity, and to delineate disease progression in the clinic? Finally, as L1CAM is not expressed in the normal colonic epithelium [166], can it provide a therapeutic handle for incurable metastatic and treatment-refractory disease?

Overall, the above findings attest to the plasticity of *Lgr5*^−^ tumour-bulk cells, suggesting it may underpin failed treatment outcomes and metastatic competence. Which differentiated/non-CSC *Lgr5*^−^ subsets are mobilized to replenish primary tumour growth, when the *Lgr5*^+^ CSC pool is compromised, and whether these are the same cell subsets that exhibit metastatic competence remains to be seen. The ability of differentiated/non-CSC *Lgr5*^−^ cells to activate a dormant plasticity program at the distant site, seed metastases, and re-establish a cellular hierarchy de novo highlights the need to target intrinsic plasticity mechanisms as well as extrinsic niche pathways in order to ablate metastatic potential.

## 15. Microenvironmental Influences on Tumour Cell Plasticity

In mouse [304] as well as human tumours [274], CSC populations are located at the base of crypt-like structures [304], near the tumour-stroma interface, suggesting that signals emanating from the stroma can impose stemness and/or induce dedifferentiation [259,261,305,306,307]. Accordingly, accumulation of nuclear β-catenin is most pronounced in tumour cells located near stromal myofibroblasts at the leading edge, correlating with CSC clonogenic potential [305] and metastatic propensity [308]. Interestingly, the membranes of cells staining positive for nuclear β-catenin, at the invasive edge, are decorated with the metastasis-initiating cell-marker L1CAM, which is also a Wnt/β-catenin target [166,309]. Furthermore, converging evidence suggests that activated CAFs elaborate a cocktail of cytokines, including HGF, OPN, SDF1, and IL17A, which stimulates Wnt/β-catenin signalling and confers enhanced clonogenic potential, therapy resistance, and metastatic competence upon nearby CSCs as well as bestowing CSC traits upon differentiated tumour cells [305,307,310,311]. Conversely, KRT20 is expressed in differentiated cells of the tumour core [274]. These findings underscore the defining influence of stromal signals in the development of aggressive CSC traits and bring forth the concept of the “migrating cancer stem cell” as a key player in metastatic competence [308].

*LGR5*-expression patterns differ between human non-serrated conventional adenomas and serrated lesions (sessile serrated adenomas/polyps and traditional serrated adenomas), likely reflecting differences in their histogenesis and their niche/TME. In conventional adenomas, expression of *LGR5* is spread throughout the gland, suggesting an expanded niche and the lack of a cellular hierarchy [312]. In contrast, serrated lesions retain basal localization of *LGR5* and display a presumptive cellular hierarchy [312]. In particular, the ectopic crypts of traditional serrated adenomas harbour basal *LGR5*^+^ cells, potentially linking the disruption of BMP gradients to the de novo generation of an ectopic niche and/or the migration of *LGR5*^+^ cells to a permissive site in response to chemotactic TME signals [87,312]. In addition, consistent with a role for LGR5 in invasion, *LGR5* expression is enriched in CD44^hi^KRT20^lo^ cells at the invasive edge of adenocarcinomas, irrespective of stage [312].

Since inflammation is a key determinant of a pro-tumorigenic environment, it is perhaps unsurprising that the inflammatory mediator PGE2 drives expansion of multiple CSC populations (expressing *LGR5*, CD133, CD44, and SOX2) and promotes liver metastasis in orthotopic tumour models [313]. Interestingly, celecoxib—a nonsteroidal anti-inflammatory drug known to inhibit prostaglandin synthesis—attenuated these effects [313], illuminating the mechanisms whereby such drugs reduce the risk of CRC. As already discussed, PGE2 also drives tumour initiation from foetal-like, regenerative *Sca1*^+^ reserve-like stem cells by stimulating the druggable PTGER4–YAP signalling axis [159]. Taken together, the abovementioned studies contend that cancer stemness is a dynamic cellular state, defined by the microenvironment rather than a particular CSC-marker phenotype or cell-of-origin.

## 16. CRC Subtypes and Niche-Signalling Pathways

Recent large-scale molecular profiling endeavours have classified CRCs into distinct subtypes with a view to relating molecular traits to clinical behaviour and therapy responses [237,314,315]. Hence, four so-called consensus molecular subtypes (CMS) have emerged from a comprehensive multipronged analysis of tumour molecular and phenotypic features (including gene expression, mutations, copy number, methylation, microRNAs, proteomics) [237]. Moreover, five CRC intrinsic subtypes (CRIS) have been proposed based on tumour cell-intrinsic transcriptional signatures, independently of any stromal contribution [315].

Hypermutated CMS1 tumours exhibit a serrated morphology and, typically, harbour *BRAF^V600E^* mutations, mismatch-repair deficiency, and CpG-island hypermethylation, leading to loss of tumour-suppressor gene function. The CMS1 TME is enriched for infiltrating immune cells (mainly TH1 and cytotoxic T cells), while also exhibiting pronounced activation of immune-evasion pathways, suggesting CMS1 tumours may be amenable to immune-checkpoint inhibitors, which restore T cell-mediated antitumor immune responses [237]. By contrast, the microenvironment of mesenchymal CMS4 tumours is proinflammatory with prominent activation of complement pathways, immune and stromal infiltration, and increased ECM deposition. In addition, CMS4 tumours are enriched for signatures associated with EMT, TGFβ signalling, angiogenesis [237], and YAP/TAZ activity [316]. Hence, CMS4 tumours carry the worst prognosis due to a heightened metastatic propensity and an inherent resistance to chemotherapy and EGFR-blockers [237,314,317]. While CMS4 tumours phenotypically and behaviourally resemble the intrinsic CRIS-B subtype, it is important to note that their elevated TGFβ levels emanate from the stroma [315,318,319,320]. In contrast, CRIS-B tumours harbour tumour cell-intrinsic deregulation of the TGFβ pathway [315]. Thus, the CRIS-B signature confers a poor prognosis in tumours with a low CAF-content and, conversely, high CAF infiltration predicts worse outcome only in non-CRIS-B tumours [315]. Nevertheless, recent works have validated the CMS-classifiers, successfully assigning multiple cell lines, primary cultures, and patient-derived xenografts to the corresponding CMS, based on their gene-expression signatures independently of stromal contribution [321].

CMS2 tumours are believed to arise via the conventional adenoma-carcinoma sequence, typically entailing aberrant activation of Wnt signalling [237]. Indeed, these tumours most resemble the CRIS-C, CRIS-D, and CRIS-E subtypes, since they are all underpinned by activated Wnt/β-catenin signalling [237,315,322]. In addition to Wnt activation, CRIS-C tumours exhibit elevated EGFR/ERBB signalling and *MYC* copy number gains, but typically retain wild-type *KRAS*, which predicts a good response to EGFR-targeted therapies [315,322]. CRIS-D tumours are enriched for amplification of Chr11p15.5, encompassing the *IGF2* locus, and exhibit activated IGF2 as well as FGFR signalling [315]. Importantly, CRIS-D tumours also express an ISC-associated gene signature, which correlates with disease recurrence [304,315]. Of note, *IGF2* and *ASCL2* are co-expressed in a subset of aggressive CRC liver metastases with Chr11p15.5 gain [323]. Consequently, therapies targeting the IGF2-receptor, IGF1R, and FGFR may be combined with LGR5-targeted regimens for CRIS-D patients. Lastly, CRIS-E tumours frequently harbour *KRAS* and *p53* mutations, rendering them refractory to anti-EGFR therapies. Interestingly, they also exhibit a Paneth cell-like gene expression profile [315,322], suggesting they may develop from metaplastic Paneth cells, well-documented in CRC histopathology [65,324]. Overall, these Wnt-driven subtypes could benefit from combinatorial strategies to perturb downstream effectors of the Wnt/β-catenin program as well as autocrine-signalling pathways, enriched in specific CRIS subgroups [63,237,315,322].

Finally, CMS3 tumours most resemble the CRIS-A subtype [315], lacking immune and inflammatory signatures and often harbouring *KRAS*-activating mutations, which confer resistance to anti-EGFR therapies [237]. Nevertheless, the so-called metabolic subtype is enriched for several metabolic pathways, including glutamine, fatty acid, and lysophospholipid metabolism, which opens up the realm of metabolic-targeted therapies [237,314].

## 17. Niche-Emancipating Mutations, Tumour Progression, and Therapeutic Implications

As discussed above, the CSC niche is pivotal in driving intra-tumoral heterogeneity within genetically homogeneous colorectal tumours. Several studies support the notion that colorectal tumours contain multiple coexisting genomic subclones, likely arising from functionally distinct CSC populations that differ in their self-renewal capacity, metastatic potential, and intrinsic chemoresistance [325,326,327,328,329]. Indeed, chemotherapy promotes clonal dominance of minor intrinsically resistant or dormant subclones [326]. Moreover, one study reported that 65% of distant and lymphatic metastases originate from independent subclones within the primary tumour, whereas only 35% derive from the same subclone [330]. Overall, clonal evolution and heterogeneity of colorectal tumours are thought to adhere to the “Big Bang” model, whereby the majority of pervasive mutations arise early during tumour development, with aggressive subclones emerging primarily when a new selective pressure—e.g., chemotherapy—is applied [331].

Engineered organoids, harbouring cooperating driver-mutations in key CRC pathways (Wnt, EGFR, p53, TGFβ, and/or PI3K), recapitulate the adenoma-carcinoma transition and exhibit a progressive loss of niche dependence, which confers a growth advantage in a hostile milieu [332,333,334,335]. While such engrafted organoids exhibit invasive features and can form micrometastases [332,333,334,335], additional molecular lesions and TME changes are required to drive colonization and metastatic outgrowth [296,336,337,338]. For example, *p53* loss allows continued proliferation and survival under stress as well as the accumulation of additional emancipating mutations that confer a competitive clonal advantage, in keeping with the Big Bang model [331,339]. The fact that overexpression of the BMP/TGFβ inhibitor NOG enables liver colonization of SMAD4-proficient *APC*^−/−^*KRAS^G12D/^*^+^*p53*^−/−^ organoids, similarly to *APC*^−/−^*KRAS^G12D/^*^+^*p53*^−/−^*SMAD4*^−/−^ counterparts, identifies the acquisition of niche independence as a key determinant of metastatic competence [339].

**Figure 4:**
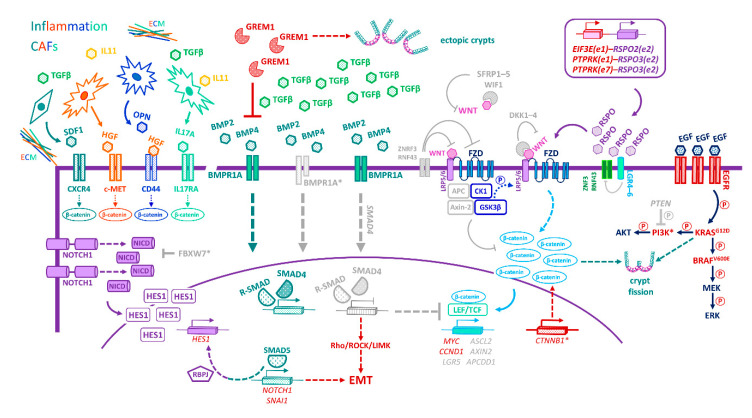
Mutations in components of niche-signalling pathways lead to “ISC emancipation” and subversion of homeostatic mechanisms. “ISC emancipation”, whereby ISCs gain autonomy from niche signalling, arises when a mutation either negates ISC dependence on pro-proliferative and pro-survival niche signals, or enables ISCs to evade growth-inhibitory stimuli. *From right to left*: Amplification of *EGFR*, activating mutations in *KRAS* (*KRAS^G12D^*), *BRAF* (*BRAF^V600E^*), or *PIK3CA* (which encodes PI3K), and *PTEN* loss-of-function mutations can stimulate MEK/ERK signalling, leading to increased proliferation and survival. The aberrant activation of Wnt signalling during CRC progression is associated with: (1) *RSPO2/3* gene fusions that elevate RSPO levels in the TME, (2) epigenetic silencing of genes encoding secreted Wnt antagonists (WNT-ligand antagonists: SFRP1–5 and WIF1; WNT-receptor antagonists: DKK1–4), (3) loss-of-function mutations in negative feedback regulators of the Wnt pathway, such as *APC*, *ZNRF3*, *RNF43*, or *Axin2*, or (4) activating mutations in *CTNNB1* (which encodes β-catenin). The pro-proliferative Wnt-target genes *MYC* and *CCND1* are typically overexpressed, whereas ISC-associated genes are often methylated in aggressive human tumours. Mutations in *KRAS* or *APC* correlate with increased crypt fission. Often found in human polyposis syndromes, disruption of BMP gradients (through overexpression of *GREM1* or the acquisition of mutations in *BMPR1A*) leads to the formation of ectopic crypts and polyps. *SMAD4* deletion/mutation and/or deregulated TGFβ signalling are further associated with niche independence, EMT, metastasis, and therapy resistance. An inflammatory drive exacerbates tumour progression, with activated CAFs and infiltrating tumour-associated populations elaborating multiple cytokines, including TGFβ, IL11, HGF, OPN, SDF1, and IL17A, which stimulate Wnt/β-catenin signalling and confer aggressive traits. Activation of Notch signalling in advanced tumours is associated with elevated levels of NOTCH1 and HES1, and inactivation of FBXW7, impairing NICD degradation. The Notch and BMP pathways synergize in a SMAD5-dependent manner to induce EMT, and BMPs activate Wnt/β-catenin signalling in the context of SMAD4-deficiency. Grey colouring indicates suppression/inactivation, multiplicity of symbols and/or red denote aberrant upregulation/activation, asterisks signify unspecified mutations, and circled P indicates phosphorylation. Solid arrows indicate direct activation, dashed arrows signify multiple intermediary steps, and lines ending with a bar denote inhibition. CAFs, cancer-associated fibroblasts; ECM, extracellular matrix; EMT, epithelial-mesenchymal transition.

Understanding how CSCs circumvent niche dependence (Figure 4) will likely inform the development of novel targeted therapies. Mutations in *APC* or *CTNNB1* lead to cell-autonomous constitutive activation of pro-proliferative Wnt signalling, setting cells along the path to niche independence. Yet, remarkably, restoring APC function to *shApcKras^G12D^p53^R172H^*^/−^ invasive carcinoma models suffices to induce cell differentiation, restore niche homeostasis, and elicit tumour regression, attesting to the therapeutic potential of targeting the Wnt pathway in CRC [336,340]. In the case of metastases, however, this differentiation therapy approach generated ectopic, functional colonic epithelium at the distant site, raising a cautionary note about possible collateral damage to the host tissue [336]. Furthermore, the development of therapies for ligand-independent CRCs that harbour constitutive Wnt-pathway activation has been fraught with the challenges of targeting complex intracellular signalling hubs as well as the toxicity associated with inhibiting the “ubiquitous” Wnt pathway [322].

Notably, *APC* and *CTNNB1* mutations are mutually exclusive with perturbations of the extracellular arm of the Wnt pathway [341]. Indeed, chromosomal translocations that increase *RSPO* expression, or mutations that block ZNRF3/RNF43-mediated ubiquitination and degradation of FZDs, augment Wnt signalling in APC-proficient tumours that therefore, crucially, retain ligand-dependence [282,342,343,344,345,346,347]. Unveiling new therapeutic vulnerabilities, these findings prompted the development of RSPO-targeted therapies for the 10% of APC-proficient colon tumours harbouring *RSPO2* and *RSPO3* gene fusions [343]. Encouragingly, antibody-mediated blockade of RSPO3 inhibited the growth of *PTPRK-RSPO3*-fusion-positive human tumour xenografts, compromising the expression of stemness genes (e.g., *LGR5*, *ASCL2*, *LRIG1*, *TERT*) and promoting differentiation [348]. Moreover, PORCN inhibitors [342,349] and anti-LRP5/6 antibodies [350] have shown promising efficacy in preclinical models of Wnt-addicted tumours. Collectively, these emergent therapies act to exhaust CSCs, downregulate CSC-associated genes, induce differentiation, and restore crypt homeostasis, thus holding promise as candidate differentiation therapeutics.

Kleeman et al. reasoned that translating these discoveries to the clinic will require a “mutation-agnostic biomarker” to stratify patients with ligand-dependent tumours [341]. Towards this aim, they profiled the differential expression of Wnt-target genes between ligand-independent and -dependent tumours. Strikingly, they found that ligand-dependent tumours tend to silence genes encoding Wnt antagonists, such as AXIN2, NKD1, APCDD1, NOTUM, and DKK4, whereas ligand-independent tumours effectively bypass Wnt-pathway negative feedback loops without the need for such selective pressure. As a result of this work, *AXIN2* hypermethylation/silencing is proposed as a tractable biomarker of ligand-dependent tumours that may help prospectively stratify patients for therapies perturbing Wnt-pathway ligands, including PORCN inhibitors [341].

Despite their tolerability, the safety of Wnt-targeted therapies remains under scrutiny, since on-target effects include diminished bone density and volume [351] as well as an unknown impact on Wnt-regulated stem cell populations in bystander organs. Safety concerns aside, the rationale of targeting Wnt signalling in the metastatic setting has further been challenged. Thus, while an *Lgr5*^+^EPHB2^+^ ISC-associated signature correlates with an increased risk of disease recurrence [304], a subset of poor-prognosis human metastatic CRCs, expressing nuclear β-catenin, harbours methylation of certain Wnt-target genes, including *LGR5*, *ASCL2*, and the negative feedback regulators *AXIN2* and *APCDD1* [352]. Accordingly, 5-azacytidine demethylation derepressed *AXIN2* and *APCDD1* and compromised xenograft growth [352]. Strikingly, while high expression of the majority of Wnt/CSC-associated genes correlated with good prognosis, the CSC population of these poor-prognosis tumours expressed an immature, embryonic stem cell-like signature, distinguished by markers of pluripotency (namely, targets of the transcriptional regulators SOX2, OCT4, and NANOG) as well as the selective methylation of Wnt/CSC-associated genes [352]. Thus, *Lgr5*^+^EPHB2^+^ ISC-associated signatures may be indicative of intestinal tissue-specific *Lgr5*^+^ ISCs as a likely cell-of-origin, whereas the immature signatures may suggest reactivation of dormant embryonic programs. Most importantly perhaps, these findings point to Wnt-independent signals regulating Wnt-target genes during the transition of adenomas to poorly differentiated, aggressive tumours. Similarly, patient-derived CD133^+^ CSCs harbour an embryonic stem cell-like signature, enriched for targets of SOX2, OCT4, and NANOG [353] as well as high HH/GLI1 signalling [353]. Here, consistent with the aforementioned findings [352], dominant-negative TCF4 enhanced metastasis despite blocking Wnt signalling in CD133^+^ CSCs [353]. Together, these studies suggest that anti-metastatic strategies should target pathways driving the immature CSC signature, e.g., HH/GLI1 signalling, rather than the Wnt/TCF axis [352,353], although Wnt agonists may also be deployed depending on the context [322,352].

Recent studies have further shown that the attenuated expression of *LGR5*, observed in advanced CRCs, often correlates with markers of TGFβ activation [354]. Indeed, LGR5 knockdown compromised TGFβ signalling and, similarly to the abovementioned studies, increased metastasis in an orthotopically implanted cell line model [354]. Here, in addition to its roles in potentiating Wnt signalling, RSPO1 was found to stimulate the formation of complexes between LGR5 and the TGFβ type II receptor, augmenting the cytostatic and cytotoxic effects of TGFβ while effectively providing a selective pressure for silencing *LGR5* during CRC progression [354]. At first, the emergent conclusion that LGR5 and/or RSPO1/LGR5 complexes function effectively as metastasis suppressors [352,353,354] may seem at odds with the finding that targeting LGR5 leads to regression of established liver metastases [297]. However, the former studies blocked LGR5-associated downstream pathways in human CSCs [352,353,354], whereas the latter study ablated entire *Lgr5*^+^ CSC subsets within engineered mouse organoid allografts [297]. Of note, it is also likely that LGR5 expression levels, isoform ratios, and functions are differentially and dynamically regulated in different CRC subtypes, disease stages, CSC subpopulations, stages of the cell cycle, and, even, distant sites [355]. Nevertheless, collectively, these studies identify repression of Wnt signalling in advanced and metastatic CRCs, contrary to the just-right levels driving adenoma formation, with the corollary that the same Wnt antagonists that retard primary tumour/adenoma growth may exacerbate metastasis [352,353,356]. Taken together with the just-right paradigm, which suggests that too much Wnt signalling can counter polyp formation [61], it has been provocatively proposed that Wnt agonists be evaluated in the metastatic setting, in combination with chemoradiotherapy and surgical resection of the primary tumour [322].

Strategies that target Wnt signalling also alter competition between ISCs with profound therapeutic implications. In mouse models, inhibition of WNT-ligand secretion (using PORCN inhibitors) [63] or RSPO-binding (using soluble RSPO-receptor ectodomains) [66] reduces the number of functional ISCs per crypt. Specifically, whereas proliferation of centre ISCs is maintained, border ISCs differentiate due to limited availability of WNT ligand/signal further from the crypt base [63]. Counter to therapeutic intent, reduction of WNT-ligand secretion allows rapid fixation of APC-deficient *Lgr5*^+^ ISCs, owing presumably to decreased competition in the ISC pool, thereby accelerating polyp formation [63]. Nevertheless, Wnt inhibition does reduce the incidence of crypt fission associated with aberrant MAPK signalling [63]. As in the metastatic setting, these data argue against employing Wnt inhibitors for APC-deficient tumours, but advocate their use for *BRAF*/*KRAS*-mutated serrated tumours that lack *APC* mutation (encompassing subsets of CMS1, CMS3, and CMS4 tumours) [63].

Although activating Notch mutations are rare, elevated levels of Notch-pathway components (*JAG1/2*, *NOTCH1*, *HES1*) are detected in human colon adenomas and adenocarcinomas [357,358]. Inactivation of FBXW7, which mediates the ubiquitination and proteasomal degradation of NICD, may conceivably underlie aberrant Notch activation in human CRCs [359]. Interestingly, active Notch signalling is associated with CRC chemoresistance [360] and metastasis [361], suggesting that its inhibition may confer therapeutic benefit in advanced, treatment-refractory disease. Constitutive activation of Notch signalling alone, however, is not sufficient to initiate intestinal adenomas. Nevertheless, in concert with *Apc* mutation, activated Notch accelerates adenoma formation in the small intestine and, additionally, causes dysplastic lesions in the colon not typically observed in mouse models, but of clear relevance to human colonic tumours [357]. Conversely, a γ-secretase inhibitor, which prevents Notch proteolytic activation, elicits differentiation of proliferative cells into post-mitotic goblet cells, stalling adenoma progression in *Apc^Min/^*^+^ mice [74]. Thus, together with Wnt signalling, the Notch pathway controls the proliferation of the undifferentiated cells that drive the growth of *Apc^Min/^*^+^ adenomas.

More recently, Notch signalling has been implicated in tumour cell plasticity. Indeed, *Notch1* marks a subset of CSCs in mouse models, typified by reduced *Lgr5* expression and activated TGFβ signalling [362]. Whether these CSCs represent the same metastasis-prone “*Lgr5*^−^TGFβ^+^” population that was discussed above [352,354] awaits further studies. Additionally, in human CRCs, Notch activity is associated with intra-tumoral heterogeneity and CSC plasticity. In xenograft models, Notch signalling promotes asymmetric division, thus yielding both fast-cycling MYC-dependent daughters (expressing LGR5, CD133, and CD44) and slow-cycling progeny (expressing BMI1, hTERT, and HOPX). These two populations can readily interconvert, reminiscent of the homeostatic and +4/reserve ISCs in the normal intestine [363]. Interestingly, Notch inhibition skews the balance in favour of *LGR5*^+^ cells, with the corollary that targeted therapies may dispel one CSC pool but enrich for another [363]. Similar conclusions can be drawn from the intra-tumoral distribution of Notch activity within human xenografts: high Notch activity demarcates centrally located epithelial tumour cells [364], whereas high Wnt/MAPK signalling is restricted to cells undergoing EMT at the tumour edge, consistent with aforementioned studies [305,308,364]. Here, inhibition of MAPK signalling had little-to-no effect on tumour growth and, instead, led to a marked increase in cells with high Notch activity. Conversely, Notch inhibition led to an enrichment of tumour cells with high MAPK activity, implicating tumour cell plasticity in therapy resistance. Nevertheless, combined inhibition of Notch and MAPK signalling compromised tumour growth more than either treatment alone, underscoring the promise of combination therapies targeting plasticity [364].

In mice, APC-deficient intestinal adenomas harbour widespread overexpression of the Notch ligand *Jag1*, consistent with expansion of the Paneth cell niche and activation of Notch signalling in *Lgr5*^+^ cells. Interestingly, deletion of *Jag1*—but not the canonical Notch effector *Rbpj*—in APC-deficient *Lgr5*^+^ cells disrupted the Paneth-cell tumour niche and compromised adenoma growth, suggesting non-canonical Notch signalling as a prospective therapeutic target [365]. Another study modelled the impact of targeting the Notch pathway in distinct CSC subpopulations. Whereas *Hes1* deletion in normal *Lgr5*^+^ or *Bmi1*^+^ ISCs compromised self-renewal without impacting homeostasis, deletion of *Hes1* in the *Lgr5*^+^ or *Dclk1*^+^ CSCs of *Apc^Min/^*^+^ adenomas elicited apoptosis, alleviating tumour burden [366]. Therefore, relatively well-tolerated γ-secretase inhibitors of Notch signalling may serve as differentiation- and apoptosis-inducing therapies for Wnt-activated CMS2 tumours.

Notwithstanding the above, *Vil1^Cre^Notch1^fl/fl^* mice spontaneously develop serrated lesions and secretory cell hyperplasia, which progress to colorectal mucinous adenocarcinomas, characterized by marked expression of proliferative (*Cdc2*, *Myc*, *Ccnb1*, *Ccne*), angiogenic (*Ang4*), proinflammatory (*Cox2*, *Hif1α*), and pro-invasive (*Areg*, *Ereg*, *Wnt5a*, *Mmp10*) genes [367]. Accordingly, *NOTCH1* mRNA expression is reduced in human colorectal mucinous adenocarcinomas, compared with non-mucinous tumours, suggesting a tumour suppressor role for Notch in these human CRC subsets [367]. Collectively, these findings reveal the context-dependent roles of Notch signalling in CRC progression and underscore the importance of stratifying tumours according to Notch-pathway activity.

Key BMP-pathway components are commonly mutated in hereditary [96,368,369,370,371] and sporadic intestinal cancers [372,373,374], where inactivation of BMP signalling correlates with adenoma-carcinoma progression. Nonsense mutations of *SMAD4* or *BMPR1A* are prevalent in familial juvenile polyposis [96,369], whereas polymorphisms or duplications upstream of the *GREM1* promoter are associated with aberrant Wnt-driven *GREM1* expression in hereditary mixed polyposis [375,376]. Both syndromes predispose to a plethora of colonic tumours. *GREM1* overexpression is also observed in the epithelium of traditional serrated adenomas, which are hence regarded as the sporadic counterparts of hereditary mixed polyposis polyps [94]. Alternatively, *BMP2* may be silenced by methylation [377]. Accordingly, reversal of *BMP2* methylation [377] or addition of recombinant BMP4 [378] induced differentiation and apoptosis, sensitizing xenograft tumours to chemotherapy [377]. Reactivation of BMP signalling may, therefore, serve as a differentiation therapy for BMP-deficient CRCs.

BMP activity is nevertheless highly context-dependent, and its interplay with the Wnt/β-catenin pathway impacts different stages of CRC progression. Early in tumour development, in a wild-type *SMAD4* context, BMP signalling functions as a tumour suppressor, inhibiting Wnt activity [379] but also suppressing stemness genes independently of Wnt/β-catenin [95]. However, loss of *Smad4* converts BMP signalling from a tumour suppressor to a metastasis promoter. As such, SMAD4-independent BMP signalling augmented Wnt/β-catenin signalling at the invasive front [379,380] and activated Rho/ROCK/LIMK kinases, ultimately promoting cytoskeletal remodelling, EMT, invasion, and metastasis [381]. Accordingly, the selective ROCK inhibitor Y-27632 suppressed liver metastasis in an orthotopic mouse model of SMAD4-deficient CRC [381]. Such approaches may thus be effective against SMAD4-deficient tumours harbouring elevated BMPR levels that presently confer a worse prognosis, compared with counterparts exhibiting low BMPR expression [381].

The effects of TGFβ signalling in CRC are similarly context-dependent, with both tumour-suppressing and -promoting roles ascribed. Whereas many CRCs harbour inactivating mutations in TGFβ-pathway components, advanced tumours often display elevated stromal TGFβ-levels. In CRC xenografts, stromal TGFβ orchestrated a pro-metastatic program: it stimulated CAFs to secrete IL11, enhancing the STAT3-dependent survival of recipient tumour cells during metastasis initiation and colonization at both liver and lung distant sites [318]. Accordingly, stromal TGFβ response signatures are predictive of disease relapse and metastasis in CRC [318], and the presence of CAFs in the TME correlates with an increased frequency of functional CSCs within tumours, a phenotype exacerbated in a TGFβ-rich stroma [319]. Notably, pharmacological inhibition of stromal TGFβ can attenuate disease progression in patient-derived tumour organoids and xenografts [319].

TGFβ is also an important instructive cue in the progression of premalignant lesions to distinct CMS subtypes [382]. Consistent with a tumour-suppressor role in conventional CMS2 CRCs, TGFβ induced apoptosis in tubular adenoma organoids (CMS2 precursors). By contrast, higher levels of TGFβ, such as those encountered in TGFβ-activated stroma, induced EMT—and not apoptosis—in *BRAF^V600E^*-mutant sessile serrated adenoma organoids, installing a mesenchymal CMS4 signature, whereas lower levels of TGFβ promoted progression to the CMS1 subtype [382]. Indeed, a TGFβ-activated stroma is a prominent feature of CMS4 xenografts, wherein elevated stromal TGFβ levels discourage T-cell infiltration, enabling tumours to evade the host immune response [237]. Consequently, TGFβ blockade evoked a cytotoxic T-cell response that prevented metastasis and sensitized established liver metastases to anti-PD1/PD-L1 therapy, highlighting the potential for combining TGFβ inhibitors with immunotherapies to curtail CMS4 progression/metastasis [337]. Similarly, while suppressed in most CRCs, BMP signalling is also enriched in mesenchymal CMS4 tumours, wherein the BMP and Notch pathways cooperate to induce an EMT phenotype, crucially deploying SMAD5 in a γ-secretase-independent manner, which renders these highly metastatic/aggressive subtypes refractory to γ-secretase inhibition [383].

Constitutive activation of NOTCH1 in the intestinal epithelium of mice, harbouring KRAS^G12D^ activation and p53 deficiency (*VilCre^ER^Kras^G12D/^*^+^*p53^fl/fl^Rosa26^N1icd^*^/+^ ; KPN), generated highly invasive, poorly differentiated, serrated intestinal adenocarcinomas that readily metastasize to the liver and recapitulate the poor-prognosis CMS4 and CRIS-B subtypes of human CRC [384]. In this first-of-its-kind autochthonous model, hyperactive NOTCH1 remodels the TME and drives the production of neutrophil chemoattractants—notably the CXCR2-ligand CXCL5 and TGFβ2—leading to the accumulation of immunosuppressive neutrophils within the pre-metastatic niche that drive immune evasion and metastasis. Targeting Ly6G^+^ neutrophil populations, using a small-molecule CXCR2 inhibitor, an ALK5 inhibitor, a TGFβ ligand-trap, or anti-Ly6G antibodies, attracted cytotoxic CD4^+^ and CD8^+^ T cells to the pre-metastatic niche and markedly reduced metastasis, providing new paths to therapy, albeit without impacting primary tumour burden [384].

Lymph node metastases of the CMS4 subtype were also described in mice, harbouring constitutive AKT1 activation and *p53* deletion, following exposure to azoxymethane [385]. In line with the KPN model, emergent *p53^ΔIEC^Akt^E17K^* tumours exhibited an immunosuppressive TME, characterized by elevated TGFβ levels and decreased T-cell infiltration, while also showing altered recruitment of multiple immune cell types. *p53^ΔIEC^Akt^E17K^* tumours also express elevated levels of *NOTCH3*, which correlates with poor patient survival and the CMS4 subtype, reinforcing the involvement of deregulated Notch signalling in this setting. Promisingly, antibody-mediated blockade of NOTCH3 markedly reduced invasion and metastasis in this model [385]. Further preclinical insights gleaned from such autochthonous, immunocompetent models will inform how epithelial tumour cell-intrinsic signalling rewires the TME and identify key stromal determinants that impact immune evasion, subtype specification, tumour progression, and therapeutic outcome while also offering new strategies to target epithelial-stromal crosstalk.

## 18. Conclusions

The pervasive plasticity of the intestinal epithelium is a double-edged sword: on the one hand, it allows the intestine to withstand constant chemical and mechanical assault and, on the other, it dooms CSC-targeted therapies to failure since depleted CSCs can be so readily replenished. Moreover, the fact that colorectal CSCs subvert the same niche-signalling pathways that sustain their normal counterparts poses a major therapeutic challenge since CSC-targeted therapies may inadvertently harm normal intestinal homeostasis or stem cells in bystander organs. In this regard, the YAP-dependent, highly plastic, foetal-like phenotype, implicated both in regeneration and cancer, holds promise as an actionable target since it is both potentially druggable [159] and without apparent detriment to homeostasis [153,386].

Developing CSC-targeted therapies that do not damage normal stem cells presents a challenge owing to the paucity of CSC-specific markers. In this respect, the identification of DCLK1 as a CSC-specific marker in *Apc^Min/^*^+^ polyps—not expressed in normal ISCs—offers hope since ablation of *Dclk1*^+^ cells (which also express *Lgr5*, CD44, and CD133) caused polyp regression, with no significant toxicity to healthy tissues [387]. Whereas this study provides proof-of-concept for targeting *Dclk1*^+^ CSCs, the recent follow-up work identifying IL17RB as a membrane marker of *Dclk1*^+^ CSCs offers a potential therapeutic handle for the subset of CRCs harbouring tuft cell-like IL17RB^+^DCLK1^+^ CSCs [388]. Moreover, the identification of L1CAM as a marker of metastasis-initiating cells, not expressed in the normal colonic epithelium, may open new therapeutic avenues for lethal metastatic disease [166].

In addition to fuelling primary tumour growth, CSCs are implicated in therapy resistance, disease recurrence, and metastasis. As such, their eradication represents a “Holy Grail” of cancer therapy. Strategies solely targeting CSCs should be wary of their inherent plasticity, which may render such therapies futile. Instead, simultaneously targeting multiple TME cues, underpinning plasticity or facilitating niche-independence, may be fruitful. Indeed, preclinical evidence supports this contention, albeit currently only for specific CRC subsets [337,342,348,349,350,384,385]. The mechanisms enabling colorectal CSCs to subvert or circumvent niche-signalling dependencies will illuminate putative targets downstream of key niche-emancipating mutations. Similarly, mechanisms empowering disseminated CSCs to navigate the metastatic cascade and harness the microenvironment, at the distant site, will inform therapeutic strategies for metastatic disease.

Important concerns remain as to whether, or not, to target Wnt signalling and in which settings/subtypes this might prove beneficial or harmful. Key to solving this conundrum will be to stratify poorly differentiated tumours, according to whether they cluster with *Lgr5*^+^EPHB2^+^ ISCs [304] or display methylation of CSC-associated Wnt-target genes [304,341], to identify patients that may benefit from Wnt antagonists or agonists, respectively. Further studies should model the relevant CRC subtypes and delineate key TME signals that instigate the epigenetic silencing of Wnt/CSC-associated genes during disease progression. Downstream effectors or epigenetic modulators, discovered thereof, may serve as prospective therapeutic targets for subtypes harbouring these immature CSC signatures [304]. In this respect, the identification of HH/GLI1 signalling as a driver of immature CSC signatures is promising [353]. Alternatively, as some of the methylated genes are negative regulators of the Wnt pathway [341], their suppression may unleash gene products that promote disease progression but offer new therapeutic targets. Epigenetic modulation is likely key to the marked plasticity of CSC (sub)populations and may serve as a combination therapy to channel CSCs towards a specific fate. Moreover, evaluating Wnt agonists for the treatment of advanced intestinal tumours will require the development of suitable subtype-specific metastatic models, which are still largely lacking.

*Lgr5*^+^ CSCs have been specifically linked to metastasis, although this does not preclude the involvement of other CSC subsets. Nevertheless, most promising is the report that ablation of *Lgr5*^+^ CSCs stunts liver colonization and causes regression of established metastases, especially since metastasis remains the leading cause of CRC-related deaths [297]. Moreover, the fact that *Lgr5*^−^ differentiated tumour cells must re-express *Lgr5* in order to repopulate the CSC pool, following its depletion during treatment [297], or to colonize the distant site [302] holds promise for ongoing efforts to develop LGR5 antibody-drug conjugates [293,355,389]. However, it will be important to determine which LGR5^−^ subsets are able to seed metastases in distant organs, and to evaluate the long-term efficacy and safety of targeting LGR5^+^ populations. Given that LGR5^+^ cells can be readily replenished, it is expected that therapy will need to be sustained for the longer term [355].

In line with the plasticity of the epithelium, multiple CSC populations have been identified within intestinal tumours, although the degree to which they overlap, or interconvert, remains unclear. It is important to note that the distribution of multiple progenitor pools along the intestinal crypt axis implies the existence of multiple spatially distinct niches, potentially governed by different molecular circuitries. In addition, basal proliferation rates, crypt dynamics, stem-cell numbers, Wnt-pathway activity and niche-signalling output, cell lineage subsets, and microbiota burden and composition vary profoundly along the length of the intestinal tract [62,137,220,261]. In tumours, such differences are further exacerbated by local variations in inflammation, hypoxia, altered ECM deposition, infiltrating cell types, and activated CAFs, all of which contribute to intra-tumoral and inter-patient heterogeneity. These considerations underscore the need to target multiple niche-signalling pathways simultaneously to block the interconversion of distinct CSC subsets within the TME.

The capacity of CSCs to dynamically interconvert or withdraw from the cell cycle, under the influence of niche signals and extraneous stresses such as chemotherapy, is a critical determinant of disease progression and therapy resistance. Importantly, evidence suggests that neoadjuvant chemotherapy enriches for a CMS4-like mesenchymal phenotype in residual tumour cell populations and liver metastases, irrespective of the original CMS designation [390]. Therefore, it is possible that CMS4-targeted therapies may be effective not only against CMS4 tumours per se, but also against the mesenchymal tumours and metastases that persist/emerge in CRC patients of any subtype after treatment with conventional chemotherapy [390].

The contributions of distinct CSC subsets to the emergence and clinical responses of specific CRC subtypes, whether the various subtypes derive from particular cells-of-origin, and whether distinct CSC populations drive primary and metastatic outgrowths are open questions in the field. The answers will inform the development of subtype-specific CSC-targeted therapies and enable clinicians to stratify patients most likely to benefit from tailored therapeutics. While this review celebrates the strides of progress in the field, much work remains to better understand the plasticity of CSCs and target their emancipation from the niche, with a view to successfully translating findings “from crypt-to-clinic”.
